# Fine-scale estimation of effective reproduction numbers for dengue surveillance

**DOI:** 10.1371/journal.pcbi.1009791

**Published:** 2022-01-20

**Authors:** Janet Ong, Stacy Soh, Soon Hoe Ho, Annabel Seah, Borame Sue Dickens, Ken Wei Tan, Joel Ruihan Koo, Alex R. Cook, Daniel R. Richards, Leon Yan-Feng Gaw, Lee Ching Ng, Jue Tao Lim

**Affiliations:** 1 Environmental Health Institute, National Environment Agency, Singapore; 2 Saw Swee Hock School of Public Health, National University of Singapore, Singapore; 3 Manaaki Whenua—Landcare Research, Lincoln, New Zealand; 4 School of Design and Environment, National University of Singapore, Singapore; 5 School of Biological Sciences, Nanyang Technological University, Singapore; Fundação Getúlio Vargas: Fundacao Getulio Vargas, BRAZIL

## Abstract

The effective reproduction number *R*_*t*_ is an epidemiological quantity that provides an instantaneous measure of transmission potential of an infectious disease. While dengue is an increasingly important vector-borne disease, few have used *R*_*t*_ as a measure to inform public health operations and policy for dengue. This study demonstrates the utility of *R*_*t*_ for real time dengue surveillance. Using nationally representative, geo-located dengue case data from Singapore over 2010–2020, we estimated *R*_*t*_ by modifying methods from Bayesian (EpiEstim) and filtering (EpiFilter) approaches, at both the national and local levels. We conducted model assessment of *R*_*t*_ from each proposed method and determined exogenous temporal and spatial drivers for *R*_*t*_ in relation to a wide range of environmental and anthropogenic factors. At the national level, both methods achieved satisfactory model performance (R^2^_EpiEstim_ = 0.95, R^2^_EpiFilter_ = 0.97), but disparities in performance were large at finer spatial scales when case counts are low (MASE _EpiEstim_ = 1.23, MASE_EpiFilter_ = 0.59). Impervious surfaces and vegetation with structure dominated by human management (without tree canopy) were positively associated with increased transmission intensity. Vegetation with structure dominated by human management (with tree canopy), on the other hand, was associated with lower dengue transmission intensity. We showed that dengue outbreaks were preceded by sustained periods of high transmissibility, demonstrating the potential of *R*_*t*_ as a dengue surveillance tool for detecting large rises in dengue cases. Real time estimation of *R*_*t*_ at the fine scale can assist public health agencies in identifying high transmission risk areas and facilitating localised outbreak preparedness and response.

## Author summary

The effective reproduction number *R*_*t*_ is an epidemiological quantity that provides an instantaneous measure of transmission potential of an infectious disease. While dengue is an increasingly important vector-borne disease, few have used *R*_*t*_ as a measure to inform public health operations and policy for dengue. This study demonstrates the utility of *R*_*t*_ for real time dengue surveillance. Using nationally representative, geo-located dengue case data from Singapore over 2010–2020, we estimated *R*_*t*_ by modifying methods from Bayesian (EpiEstim) and filtering (EpiFilter) approaches, at both the national and local levels. We conducted model assessment of *R*_*t*_ from each proposed method and determined exogenous temporal and spatial drivers for *R*_*t*_ in relation to a wide range of environmental and anthropogenic factors. At the national level, both methods achieved high accuracy, but disparities in performance were large at finer spatial scales when case counts are low. This study demonstrates the potential of *R*_*t*_ as a dengue surveillance tool for detecting large rises in dengue cases. Real time estimation of *R*_*t*_ at the fine scale can assist public health agencies in identifying high transmission risk areas and facilitating localised outbreak preparedness and response.

This is a *PLOS Computational Biology* Methods paper.

## 1. Introduction

Dengue is an arboviral disease that imposes significant health and economic burdens across the globe [1]. It is transmitted by *Aedes* mosquitoes, primarily the highly urban-adapted vector *Aedes aegypti* [2]. Increased levels of urbanization, human population density and climate change have led to an expanded geographical coverage of the primary vector *Ae*. *aegypti*, resulting in an estimated annual 105 million dengue infections globally [3]. The burden of dengue is high, particularly in the tropics and subtropics where vector breeding conditions are favourable, and transmission persists year-round [4–6].

Located within Southeast-Asia, Singapore is a highly urbanised island city-state with a tropical climate conducive for *Aedes* mosquito breeding and dengue transmission [7]. Singapore faces regular dengue outbreaks with all four dengue serotypes co-circulating all year round [8]. Although dengue vaccine, Dengvaxia (CYD-TDV), has been made available commercially since 2017, its efficacy against different serotypes varies and administering it may increase the risk of severe dengue disease in individuals who have not been previously infected. The vaccine is thus safe for use only on individuals with previous dengue infections [9]. As a result, community-based vector control remains the key strategy to mitigate dengue transmission in Singapore [10]. However, vector control activities are often resource and labour intensive, and critically require early warning systems for pre-epidemic preparedness and efficient vector control deployment [11].

While forecast models have been developed to pre-empt dengue outbreaks in Singapore [12], these tools remain a challenge to implement for real-time policy advice as the data needed to parameterize and feed the model are often difficult or impossible to obtain immediately. Furthermore, these tools do not provide insights on the current transmissibility of dengue as well as the efficacy of vector control measures, both of which are important to policymakers. The effective reproduction number, which is a measure of disease transmission intensity, requires only the input of case data and an estimate of the generation interval distribution [13], might therefore serve as an attractive complement to more complicated forecasting models.

More formally, the effective reproduction number, *R*_*t*_, is an important epidemiological quantity that provides an instantaneous measure of the transmission potential of an infectious disease [14]. It is defined as the expected number of new infections caused by an infectious individual over the course of its infectious period, in a population composed of both susceptible and non-susceptible individuals. *R*_*t*_ is different from the basic reproduction number (*R*_0_), which is the expected number of secondary infections from a primary case in a population where every individual is susceptible. During the course of an epidemic, individuals become infected or immune and are no longer susceptible, the estimation of *R*_*t*_ is therefore more appropriate [15]. When *R*_*t*_ *>* 1, we can expect an increase in the number of cases and a growing outbreak, whereas sustained *R*_*t*_ *<* 1 signifies that the epidemic is waning and likely to enter a more controlled phase [16]. *R*_*t*_ estimates have been used to nowcast the rates of spread of various infectious diseases such as yellow fever [17] and severe acute respiratory syndrome (SARS) [18], as well as to quantify the efficacy of disease control measures [18]. During the COVID-19 pandemic, *R*_*t*_ estimates were especially valuable in providing up-to-date synopses of global transmission and evidencing the impact of control actions such as social distancing and lockdowns [19].

There are three primary approaches for estimating *R*_*t*_; (1) Forward-looking, (2) Backward-looking and (3) Backward & Forward looking. Developed by Wallinga and Teunis [20], the forward-looking method is based on the probabilistic reconstruction of transmission trees and on counting the number of new cases per infected individual. It computes the case reproduction number, *R*_*c*,*t*_, which is a function of *R*_*t*+*j*_ for future time *j*≥0. This method requires incidence data beyond time *t* for its estimate, hence it is suited for retrospective analyses [13]. The backward-looking method, also known as the *Cori et al* (EpiEstim) method [14], computes the instantaneous reproduction numbers by inferring how past infections propagate to form the observed incidence at time *t*. This method only requires incidence data prior to time *t*, hence it is suited for real-time investigation [21]. EpiNow2, a substantial extension of EpiEstim was recently introduced [22]. The key advantage of EpiNow2 over EpiEstim is that it accounts for delays in reporting and estimates *R*_*t*_ even when recent data is incomplete, nonetheless, this comes at a significant computational cost. However, these methods have several limitations. Estimates from the forward-looking method are right censored when *t* is near the last observed time point [13]. The backward-looking method, on the other hand, suffers from edge effects when *t* is close to the first observed time point [14]. Estimates near the start and end of the incidence time series are therefore not reliable under the backward-looking and forward-looking methods respectively. Furthermore, in period with low incidences, these methods produce estimates of *R*_*t*_ which are driven by assumptions of the prior distributions of *R*_*t*_ rather than the likelihood of disease case counts, resulting in unreliable estimates [14]. The backward & forward looking method, also termed as EpiFilter [23], was thus developed to ameliorate some of these limitations. This method unifies the backward-looking and forward-looking methods, integrating both forward and backward looking information to compute *R*_*t*_, nullifying the edge-effect problems experienced by the former methods. In addition, the method makes minimal prior assumptions for *R*_*t*_, allowing it to handle periods where recorded case counts are scarce. While these methods provide useful estimators of disease transmissibility, modifications are necessary for application to vector borne diseases such as dengue, whose transmissibility depends on extrinsic factors such as the vector life cycle, weather variations and vector control [24,25]. As a result, *Codeco et al* developed a method for constructing a temperature-dependent generation interval, which accounts for the influence of temperature on the extrinsic incubation of dengue, for estimating the effective reproduction number [26].

In this paper, we explored the utility of effective reproduction numbers for real-time dengue surveillance nationally and on the local level. First, we nested estimation of dengue generation intervals comprising both the intrinsic and extrinsic stages of infection together with effective reproduction number estimation under two methods, EpiEstim and EpiFilter. This was done at both the national and local level, using spatially resolved daily dengue case data in Singapore. Next, we evaluated and compared the estimated *R*_*t*_ and model fit produced from both methods at both temporal resolutions. Lastly, we determined the temporal and spatial drivers for the estimated national and local level effective reproduction numbers.

## 2. Materials and methods

### Data

Dengue is a notifiable disease under the Infectious Diseases Act in Singapore, where all notifications of laboratory-confirmed cases to the Ministry of Health are legally mandated. We obtained daily reports of all dengue infections aggregated by date of onset of illness from 2010 to 2020 from the Ministry of Health, Singapore. All laboratory-confirmed dengue cases were anonymized, that is removal of sensitive patient information, prior to analysis. We divided the residential area of Singapore into small spatial units (n = 1242), each with an average area of 0.09827 *km*^2^ and containing a cluster of residential blocks bounded by roads, and obtained the daily reports of dengue cases for each spatial unit.

Using spatial data mainly from 2018, we obtained the percentage cover of each land cover type from the WorldView and QuickBird satellites [27] and aggregated them according to the respective spatial unit. The land cover types included (i) freshwater, (ii) impervious, (iii) non-vegetated pervious surfaces, (iv) vegetation with structure dominated by human management (with tree canopy), (v) vegetation with structure dominated by human management (without tree canopy), (vi) vegetation with limited human management (with tree canopy) and (vii) vegetation with limited human management (without tree canopy). Population-based statistics were provided by the Urban Redevelopment Authority, Singapore.

We obtained local climate data from 11 mainland weather stations located across the study area from the Meteorological Services Singapore (MSS) from 2009 to 2020. We used the arithmetic mean of climate data across all stations to derive daily measures of mean, maximum and minimum ambient temperature, relative humidity and rainfall. We derived daily measures of absolute humidity from measures of mean temperature and relative humidity [28].

### Modelling overview

Below, we describe modifying the EpiEstim [14] and EpiFilter [23] approaches for estimating effective reproduction numbers for dengue while simultaneously nesting uncertainty in the dengue generation intervals. This was conducted by **(A)** estimating effective reproduction numbers nationally using an aggregated time series of dengue case counts and **(B)** on the local level, comprising spatial units as described in the preceding section. We compared the estimated effective reproduction numbers between each method and examined whether they can perform well in providing realistic estimates at both spatial resolutions. Model fit was also assessed under three model assessment criteria at both scales. Lastly, we determined the temporal and spatial drivers for the estimated national and local level effective reproduction numbers by posthoc looking at relevant associations between the effective reproduction numbers and temporal/spatial covariates using a regression approach.

### Estimation of effective reproduction numbers

Define *I*_*s*_ as the daily number of newly reported dengue cases at time *s* and a Poisson distribution is used to characterize the reproductive dynamics of infectious disease transmission with *I*_*s*_~*P*_*o*_(*R*_*s*−1_Λ_*s*_). Where *R*_*s*_ is defined as the average number of secondary cases at time *s*+1 that one primary case at *s* infects (i.e, the effective reproduction number), while $\Lambda_{s}=\sum_{u=1}^{s}I_{s-u}w_{u}$ is the infection potential of the disease up to time *s*−1 and summarizes how previous cases contribute to upcoming cases at time *s*. *w*_*u*_ is the probability that it takes *u* time units for a primary case to infect a secondary case. As the time of infection is difficult to recover, we follow [14] by approximating *w*_*u*_ with the serial interval, i.e the corresponding times of symptom onset. Both the infection dynamics of human and mosquito populations affect the infection potential of dengue. Following [29], we denote:

$$s_{m_{e}\to m_{i}}=\theta_{m}+{\mu_{m}+c}_{m}$$ (1)

$$s_{m_{i}\to m_{d}}=\mu_{m}+c_{m}$$ (2)

$$s_{h_{d}\to h_{i}}=\theta_{h}+\mu_{h}$$ (3)

$$s_{h_{i}\to h_{r}}=\alpha_{h}+\mu_{h}$$ (4)

as the rates for an exposed mosquito to transition into an infective state $S_{m_{e}\to m_{i}}$ followed by removal in the population $S_{m_{i}\to m_{d}}$ and the rate for an exposed individual human to transition into an infective state $S_{h_{e}\to h_{i}}$ followed by recovery $S_{h_{i}\to h_{r}}$. *θ*_*m*_, *θ*_*h*_ refer to the extrinsic and intrinsic incubation rates respectively. *c*_*m*_ the control effort rates, *μ*_*m*_, *μ*_*h*_ the mortality rates for mosquitoes and hosts respectively, and *α*_*h*_ the recovery rate for hosts. Table 1 shows the range of values used for each of the parameters. These range of estimates were obtained from literature. Furthermore, due to the limited geographic range and weather variability in Singapore, we did not assume these estimates to vary spatially/based on climate.

**Table 1:** Parameters used in estimating the generation interval distribution.

Parameter	Biological meaning	Range of values
*μ*_*m*_	Average mosquito mortality rate	0.000–0.200 day^-1^ [30]
*θ*_*m*_	Extrinsic incubation rate	0.067–0.500 day^-1^ [31]
*μ*_*h*_	Human mortality rate	0.000033–0.000034 day^-1^ [32]
*θ*_*h*_	Intrinsic incubation rate	0.100–0.330 day^-1^ [31]
*α*_*h*_	Recovering rate	0.143–0.500 day^-1^ [33]
*c*_*m*_	Control effort rates	0–1 [29]

We can approximate the generation interval distribution as the combination of four exponential distributions [34] as parameterized by the above $\left\{s_{m_{e}\to m_{1}}{exp}^{-t\times s_{m_{e}\to m_{i}}},\;s_{m_{i}\to m_{d}}{exp}^{-t\times s_{m_{i}\to m_{d}}},\;s_{h_{e}\to h_{i}}{exp}^{-t\times s_{h_{e}\to h_{i}}},\;s_{h_{i}\to h_{r}}{exp}^{-t\times s_{h_{i}\to h_{r}}}\right\}$ and can be given by:

$$w_{u}=\sum_{j=\{m_{e}\to m_{i},m_{i}\to m_{d},h_{e}\to h_{i},h_{i}\to h_{r}\}}\frac{\prod_{i\neq j}s_{i}exp(-s_{j}t)}{\prod_{j=1,j\neq i}\left(s_{j}-s_{i}\right)}$$ (5)

The effective reproduction number *R*_*s*_ is important for nowcasting and forecasting the overall epidemic trajectory of an infectious disease and yields convenient interpretations. If *R*_*s*_ *>* 1 then we can expect the epidemic trajectory to grow, as the number of infections increase monotonically with time, whereas if *R*_*s*_ *<* 1 is sustained, we can consider the epidemic as being controlled and will eventually be eliminated [16]. Following [14], to enhance the reliability of *R*_*s*_ estimates, we assume stable epidemic transmission properties over a sliding window of size *k*, defined at the time *s* as *t*(*s*) *ϵ* {*s*, *s*−1,…,*s*−*k*+1}. Let the effective reproduction number over this window be *R*_*τ*(*s*)_ and we apply the conjugate gamma prior distribution for this parameter following [14]:

$$R_{\tau (s)}∼Gamma(a,\frac{1}{c})$$ (6)

with *a* = 1 and *c* = 2 as the shape and scale hyperparameters respectively. We yield the following gamma posterior distribution for *R*_*τ*(*s*)_ which can easily be sampled from, given the relevant window of past incidence curve data

$$R_{\tau (s)}|l_{\tau (s)}∼Gamma\;\left(\alpha_{\tau (s)}=a+I_{\tau (s)},\beta_{\tau (s)}=\frac{1}{c+\lambda_{\tau (s)}}\right)$$ (7)

where *l*_*τ*(*s*)_ = ∑_*u*∈*τ*(*s*)_*I*_*u*_ and *λ*_*τ*(*s*)_ = ∑_*u*∈*τ*(*s*)_Λ_*u*_. We then yield the posterior mean estimate of the effective reproduction number $E[{\hat{R}}_{\tau (s)}|l_{\tau (s)}]=\alpha_{\tau (s)}\beta_{\tau (s)}$ by taking the mean of the gamma distribution (7).

Given the serial interval distribution *w*_*s*_, past data on the total number of incident cases *I*_0:*t*−1_ and the reproduction number *R*_*t*_ at time *t*, we have the expected number of incident autochthonous cases by definition:

$$E(I_{t}|I_{0:t-1},\;w_{s},\;R_{t},\;X_{t})=R_{t}\Lambda_{t}$$ (8)

A Poisson generative distribution for the number of local cases at time step *t* is assumed, with the probability of observing $I_{t}^{local}$ cases at *t* being:

$$P(I_{t}|I_{0:t-1},\;w_{s},\;R_{t},\;X_{t})∼P_{o}(R_{t}\Lambda_{t}(w_{s}))$$ (9)

The baseline reproduction number is assumed to be constant [following 14] over the time period [*t*−*τ*,*t*] where *R*_*t*_ is estimated. The probability of observing the local incidence *I*_[*t*−*τ*,*t*]_ given *R*_*t*_ and lagged incidence data *I*_[0:*t*−*τ*−1]_ is given by:

$$P(I_{t-\tau :t}|I_{0:t-\tau -1},\;w_{s},\;R_{t},\;X_{t})=\prod_{k=t-\tau }^{t}\frac{{\left(R_{t}\Lambda_{t}(w_{s})\right)}^{I_{k}}exp(R_{t}\Lambda_{t}(w_{s}))}{I_{k}!}$$ (10)

### Effective reproduction numbers under low case counts

The above method can be used for endemic diseases such as dengue, where cases counts are consistently above zero at the national scale. Subdivision of case counts to the local level, where interventions are more likely to take place, means that there will be periods where case counts are likely to be near zero. Consider the case where *I*_*τ*(*s*)_ = λ_*τ*(*s*)_ = 0, then following (7), the posterior mean estimate for the effective reproduction number is the prior: $R_{\tau (s)}=\frac{a}{c}$. Therefore, when there are few/no case counts to constrain the posterior distribution, estimates of effective reproduction numbers via the method of [14] are driven by prior distributions and may be unreliable at the local level.

We therefore follow [23] by adapting the Bayesian recursive filter to estimate *R*_*s*_. In summary, the effective reproduction number *R*_*s*_ is taken as a hidden state to be inferred, with the dynamic evolution characterized by the following state equation:

$$R_{s}=R_{s-1}+(\eta \sqrt{R_{s-1}})\epsilon_{s-1}$$ (11)

Where *R*_*s*_ depends dynamically on the previous state *R*_*s*−1_, *η* some free parameter controlling the correlation among successive instantaneous reproduction numbers and ensures that *R*_*s*_ is non-negative, and *ϵ*_*s*−1_~*N*(0,1) is the white noise term, characterized by a standard normal distribution. Estimation of *R*_*s*_ then proceeds by recursive filtering, which consists of two separate prediction and filtering steps.

First, the prediction step constructs a sequential prior predictive distribution prediction. This allows estimation of the effective reproduction number at the current time point s given past data $I_{1}^{s-1}$ and the previous state *R*_*s*_:

$$\begin{array}{c} p_{prediction}=P(R_{s}|I_{1}^{s-1})\\ =\int P(R_{s}|R_{s-1},\;I_{1}^{s-1})p_{s-1}dR_{s-1}\\ p_{s}\propto P(I_{s}|R_{s},\;I_{1}^{s-1}) \end{array}$$ (12)

where $P(R_{s}|I_{1}^{s-1})∼N(R_{s-1,}\eta^{2}R_{s-1,})$ following the state equation in (11), $P(I_{s}|{R_{s},\;I}_{1}^{s-1})$ the observation Eq (8), and $p_{s}=P(R_{s}|I_{1}^{s})$ the posterior filtering distribution. Note here that we modify the prediction step to incorporate uncertainty in the generation interval of dengue through (8). Approximation of *p*_*prediction*_ follows the EpiFilter procedure as detailed in [23].

Next, the recursive smoothing step is conducted to update past estimates of *R*_*s*_ as new data accumulates. Specifically, consider the filtering distribution ***p***_*s*_ and predictive distributions $p_{s+1}=P(R_{s+1}|I_{1}^{s})$ which are obtained from (12) to obtain $q_{s}=P(R_{s}|I_{1}^{t})$, the smoothing posterior distribution is given by [23]. $q_{s}=P(R_{s}|I_{1}^{t})$ provides the posterior distribution of *R*_*s*_ given compete information on reported case counts $I_{1}^{t}$:

$$q_{s}=p_{s}\int P(R_{s+1}|R_{s},\;I_{1}^{s})q_{s+1}p_{s+1}^{-1}dR_{s+1}q_{s}\propto r_{s}p_{s}P{\left(R_{s}\right)}^{-1},\;if\;r_{s}\approx P(R_{s}|I_{s+1}^{t})$$ (13)

The equation is solved by noting that *q*_*t*_ = *p*_*t*_ and iterating backwards in time to obtain the first smoothing distribution ***q***_1_. The integrals are approximated using sums over the grid *R* and distributions are *m* element vectors. Eq (13) sequentially updates our earlier filtering solutions to include future data and forms the second half of EpiFilter.

The estimated number of case counts was obtained using the conditional mean function as follows:

$${\hat{I}}_{s}=E(I_{t}|I_{0:t-1},\;w_{s},\;R_{t},\;X_{t})={\hat{R}}_{t}\Lambda_{t}$$ (14)

and was assessed for model fit under the coefficient of determination, mean squared error (MSE) and mean absolute scaled error (MASE). The coefficient of determination is a goodness-of-fit measure that quantifies the proportion of variation in the observed case counts that is explained by the estimated case counts. It is represented as a value between 0.0 and 1.0, with 1.0 indicating a perfect fit and 0.0 indicating that the estimated case counts fail to accurately model the observed case counts. MSE, on the other hand, is an accuracy metric that measures how close the estimated case counts are to the observed case counts. It is defined as the average squared difference between the estimated and observed case counts. MSE is always a positive value, with lower MSE indicating higher accuracy. Lastly, MASE compares the one-step ahead predicted cases counts using the *R*_*t*_ framework to the output of a one-step naïve forecasting approach, which equates the one-step ahead forecast for time *t*+1 to the observed case count at time *t* (*i*.*e*. ${\hat{y}}_{t+1}=y_{t}$). MASE provides an indication of forecast accuracy for the *R*_*t*_ framework, with values greater than 1.0 indicating that forecasts from the one-step naïve forecast outperforms forecasted case counts derived from the *R*_*t*_ framework, and vice versa.

### Determining temporal and spatial drivers for effective reproduction numbers

Denote ${\hat{R}}_{t\times 1}$ as the estimated reproduction number over all timepoints under either method described in the preceding two sections. We determined post-hoc whether there are any additional associations between *p* ecologically relevant factors ***X***_*t*×*p*_ and estimated effective reproduction numbers on the national and local level by estimating the following regression:

$$\hat{R}=X\beta +\varepsilon$$ (15)

where *β*_*p*×1_ refers to the vector of regression coefficients of interest as estimated using the LASSO framework. The LASSO framework was used due to the large number of potentially relevant temporal covariates with a large number of lags (*p* = 120). Briefly, ***X***_*t*×*p*_ consists of factors such as climate and dengue case counts of up to two weeks lags so that possibly long term associations can be detected. Dengue case counts were included to control for the impact of case numbers on estimated reproduction numbers, so that the residual impact of other covariates on *R*_*t*_ may be delineated. LASSO was used to account for possible multicollinearity and allow for variable selection of important factors. Ten-fold cross validation was first conducted to yield test error rates which do not suffer from unreasonably high bias or variance [35]. The cross-validation step optimizes the regularization parameter *λ* using deviance as the tuning criterion. We then refitted our data using the optimal regularization parameter λ* to obtain the optimal *β*. Uncertainty in $\hat{R}$ was accounted for by taking each drawn sample of $\hat{R}$ under their respective estimation procedure as a separate dataset for which *β* was estimated. 95% uncertainty intervals for the regression coefficients were obtained by taking the 2.5% and 97.5% quantiles of the nested group of regression coefficients as obtained from each sample under their respective estimation procedure.

Spatial covariates considered were population density, premise type and the percentage cover of the various land cover types described in the data subsection. These spatial covariates did not vary substantially across timepoints (See S1 Fig). We aggregated the effective reproduction number as features such that *ξ*_1*xj*_ = *f*(***r***_*t*×*j*_), where we denote the effective reproduction number as estimated in each locale as ***r***_*t*×*j*_. Where *f* is a function aggregating the estimated reproduction number at any locale to some summary statistic for the location, which does not vary with time. We tried two aggregation functions, the mean ***r***_*t*×*j*_ at a location and the percentage of time ***r***_*t*×*j*_>1.0. Moran’s I was first used to test for spatial autocorrelation in the aggregated summary statistics of the effective reproduction number [36]. We next examined the association between the spatial covariates and these aggregated summary statistics using generalized linear models, taking the aggregates as the dependent variable and independent variables being the spatial covariates. Backward elimination was used to obtain the most parsimonious final model.

## 3. Results

### Effective reproduction numbers at the national and local level

**Fig 1:**
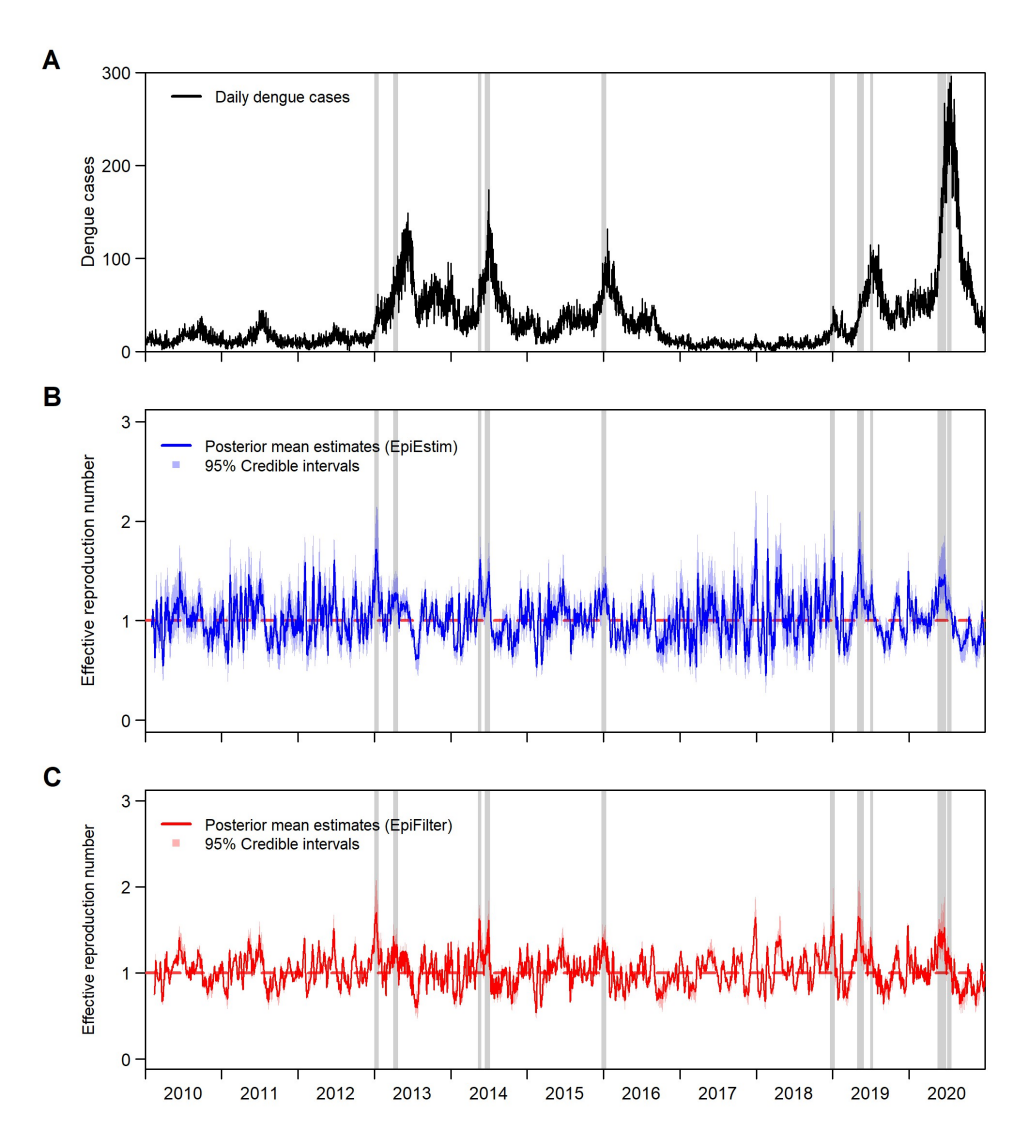
(A) Daily dengue case counts from 2010 to 2020 (B) Estimated daily effective reproduction number from 2010 to 2020 under EpiEstim method (C) Estimated daily effective reproduction number from 2010 to 2020 under EpiFilter method. Shades represent the 95% credible intervals. Grey shades represent sustained periods of high transmissibility (i.e having at least 14 days of *R*_*t*,*EpiEstim/EpiFilter*_ *>* 1.0).

A total of 137,712 dengue cases were reported over the study period, with an average of 34.3 dengue cases reported per day. Reported case counts by date of onset demonstrate that low levels of dengue cases were found in 2010–12 and 2015–18 at an average of 18.0 dengue cases per day, while elevated levels of dengue cases are found in 2013–14 and 2019–20 at an average of 62.8 dengue cases per day (Fig 1A). The posterior mean estimates of the effective reproduction number derived using EpiEstim (*R*_*t*,*EpiEstim*_) and EpiFilter (*R*_*t*,*EpiFilter*_) methods were presented in Fig 1B and 1C, along with its 95% credible interval. *R*_*t*,*EpiEstim*_ estimates ranged between 0.45 and 1.82, with a median value of 1.01 across 2010 to 2020, while *R*_*t*,*EpiFilter*_ estimates ranged between 0.54 and 2.91, with a median value of 1.02. These demonstrate that dengue in Singapore hovered between controlled and growing outbreak phases of transmission. By defining sustained periods of high transmissibility, as having at least 14 days of *R*_*t*,*EpiEstim/EpiFilter*_ being greater than 1.0, we note that *R*_*t*,*EpiEstim/EpiFilter*_ was able to detect large rises in dengue case counts in the study setting. This was apparent by observing the sustained periods of high transmissibility prior to outbreaks in 2013, 2014, 2019 and 2020 (Fig 1B and 1C). The 14-day threshold was based on the dengue case clustering criteria in Singapore, which is derived by adding the mean intrinsic and extrinsic incubation period of dengue [37].

**Fig 2:**
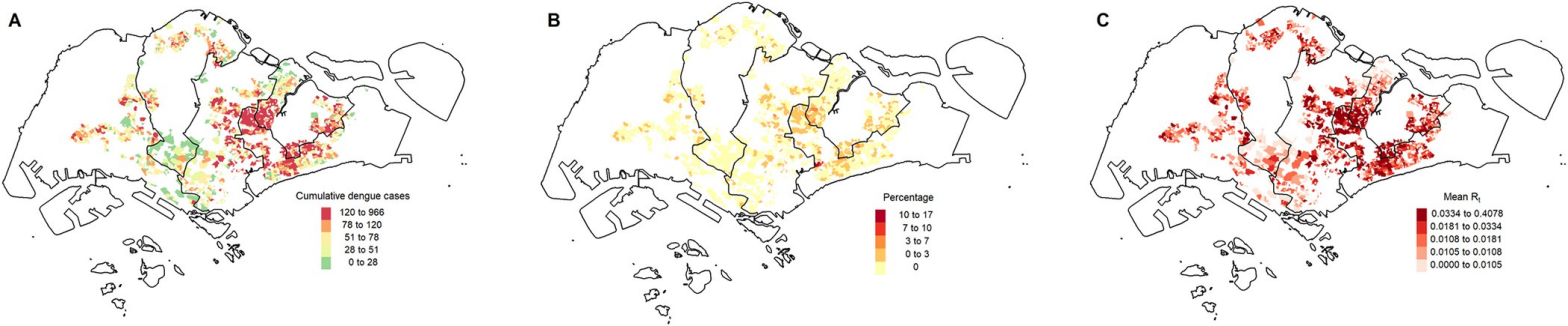
(A) Cumulative reported dengue case counts (B) Percentage of time *R*_*t*,*EpiFilter*_ *>* 1.0 (C) Mean *R*_*t*,*EpiFilter*_ from 2010 to 2020 across spatial units in Singapore. The figure was created with base layer obtained from https://gadm.org/maps.html.

The geographic distribution of dengue cases was depicted in Fig 2A. The eastern regions of Singapore had the cumulative highest case burden. Due to the small size of each spatial unit, case counts were low. Each spatial unit had zero reported dengue case counts 98.2% of the time on average, with the average number of reported dengue cases per spatial unit per day being 0.02 (Range: 0–16). However, each spatial unit had on average over 80.1 cases (Fig 2A, Range: 1–966) over the period of 2010 to 2020 cumulatively. The posterior mean estimates of the effective reproduction number derived using EpiFilter method (*R*_*t*,*EpiFilter*_) were presented in Fig 2B and 2C. Mean *R*_*t*,*EpiFilter*_ was on average 0.023, with the mean percentage of time *R*_*t*,*EpiFilter*_ being above 1.0 at 0.21% across spatial units across the study period. Both the percentage of time *R*_*t*,*EpiFilter*_ was above 1.0 and mean *R*_*t*,*EpiFilter*_ followed the case burden closely, with higher values concentrated in the eastern regions.

### Model assessment at the national and local level

At the national level, we obtained the expected case counts using (14) and assessed model fit using three metrics, namely, the coefficient of determination, mean square error and mean absolute scaled error under both proposed methods for estimating effective reproduction numbers. The model fit is satisfactory, as evidenced by the high adjusted R-squared value (Table 2: $R_{EpiEstim}^{2}$ = 0.95, $R_{EpiFilter}^{2}$ = 0.97), low mean square error (Table 2: *MSE*_*EpiEstim*_ = 75.2, *MSE*_*EpiFilter*_ = 36.2) and mean absolute scaled error which are less than 1 (Table 2: *MASE*_*EpiEstim*_ = 0.70, *MASE*_*EpiFilter*_ = 0.53). This demonstrates that both methods work well in fitting dengue case count data over the study period and were outperforming the one-step naive forecasts.

**Table 2:** Model assessment and spatial autocorrelation metrics under the EpiFilter and EpiEstim methods. Bolded numbers reflect superior model performance under the respective model assessment metric.

Metric	EpiFilter^1^	EpiEstim^1^	EpiFilter^2^	EpiEstim^2^
Adjusted *R*^2^	**0.97**	0.95	0.08	**0.09**
MSE	**36.2**	75.2	**0.02**	0.03
MASE	**0.53**	0.70	**0.59**	1.23
Moran’s I (Mean *R*_*t*_)^3^				
Test Statistic (p-value)			0.46 (0.00)	0.71 (0.00)
Moran’s I (% *R*_*t*_>1.0)^3^				
Test Statistic (p-value)			0.38 (0.00)	0.58 (0.00)

^1^Nationally

^2^Average across spatial units.

**Fig 3:**
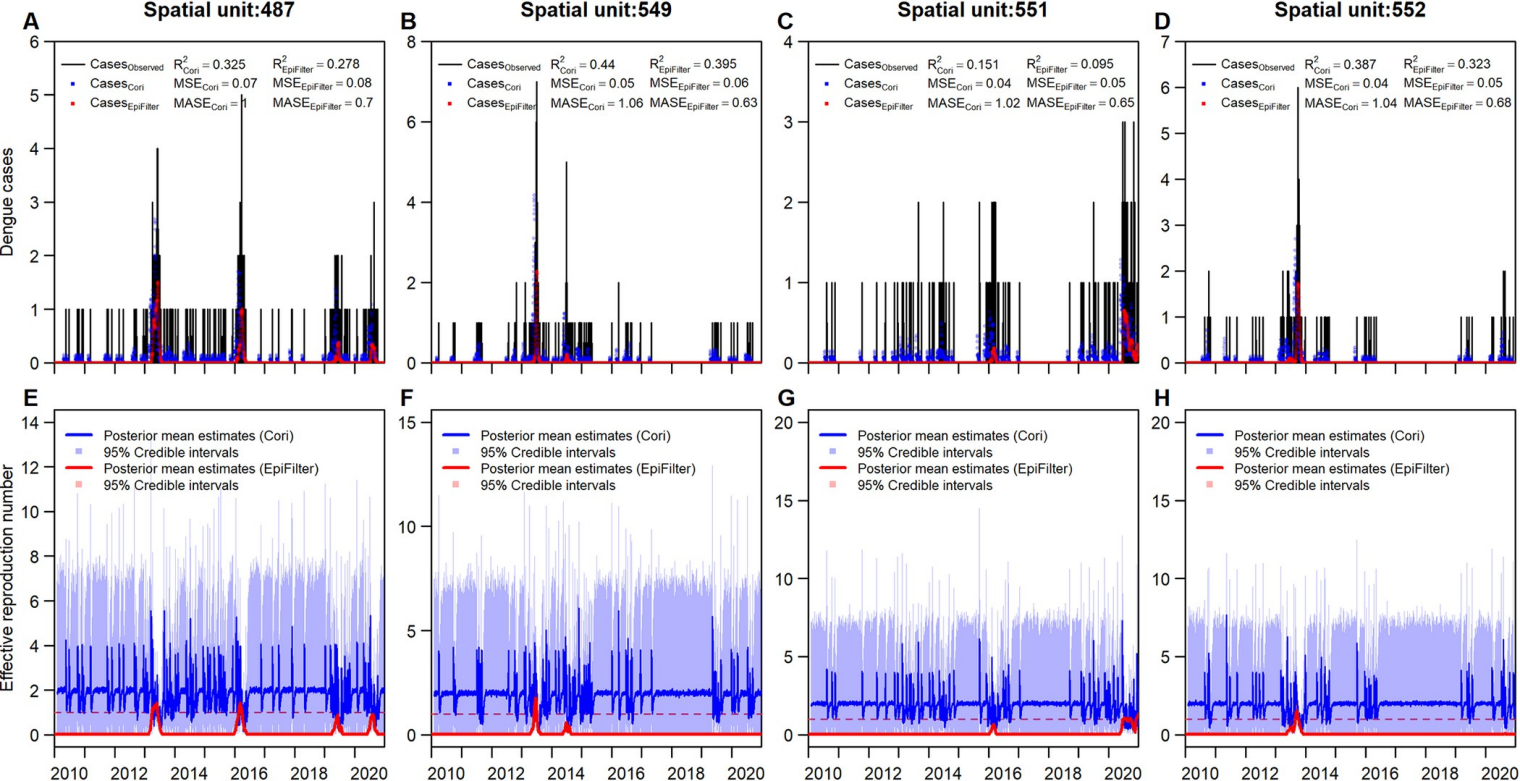
(A-D) Daily reported dengue case counts in 4 spatial units (E-H) Estimated effective reproduction numbers under EpiEstim and EpiFilter methods in 4 spatial units.

We examined the daily reported case counts and posterior mean estimates of the effective reproduction number calculated across each spatial unit to compare the EpiFilter and EpiEstim methods. The EpiFilter method yielded an average adjusted R-squared value of 0.08 and an average mean absolute scaled error of 0.59. In contrast, EpiEstim method had an average adjusted R-squared value of 0.09 and an average mean absolute scaled error of 1.23. The mean squared error was also lower for the EpiFilter method but marginally so (Table 2: *MSE*_*EpiEstim*_ = 0.03, *MSE*_*EpiFilter*_ = 0.02). Both methods had a low adjusted R-squared value due to the large number of null reported case counts in each spatial unit over the study period. However, estimation under the EpiFilter method outperforms the EpiEstim method, yielding lower mean squared error and mean absolute scaled error when averaged across all spatial units (Table 2). EpiFilter also does not allow prior distribution assumptions to take over under periods of low case counts for each spatial unit, providing more realistic estimates of effective reproduction numbers, and thus model fit under this scenario (Fig 3 and S1 Appendix).

### Associations between effective reproduction numbers and ecological confounders

The LASSO framework was used to identify the temporal drivers of the estimated national *R*_*t*,*EpiEstim*_. Among the covariates, lagged reported dengue case counts and sporadic case counts were associated with the estimated *R*_*t*,*EpiEstim*_. Shown in Table 3, increases in dengue cases were associated with an immediate and delayed rise in *R*_*t*,*EpiEstim*_ of up to 6 days. However, the association was reversed from the 8th to 14th day, resulting in a reduction in *R*_*t*,*EpiEstim*_ (Table 3). Similarly, sporadic cases with lags of 0–6 days were positively associated with *R*_*t*,*EpiEstim*_ while sporadic cases with lag of 7–14 days were negatively associated with *R*_*t*,*EpiEstim*_ (Table 3). Controlling for dengue case counts across time in the same regression, we found that none of the included climate variables were associated with the estimated *R*_*t*,*EpiEstim*_ (Table 3).

**Table 3:** Regression coefficients and associated 95% uncertainty intervals in parenthesis as estimated under LASSO, with intervals obtained from 5000 bootstrap samples and dependent variable being *R*_*t*_ *× 1000 estimated under the EpiEstim* framework.

Lag^1^	Cases^2^	Sporadic Cases^3^	Mean AH^4^	Mean T^5^	Max T^6^	Min T^7^	Rainfall^8^
**0**	2.27 (1.48, 3.75)*	2.41 (0.59, 3.61)*	3.2 (0, 7.85)	0.11 (0, 1.33)	-2.76 (-7.03, 0)	-0.02 (-3.03, 3.35)	-0.15 (-0.45, 0)
**1**	1.48 (1.14, 2.05)*	2.53 (1.27, 3.45)*	2.39 (0, 7.17)	0.03 (0, 0.03)	-1.5 (-5.46, 0)	-0.33 (-3.37, 1.57)	-0.17 (-0.48, 0)
**2**	1.53 (1.22, 1.96)*	2.24 (1.16, 3.07)*	0.91 (0, 4.96)	0.06 (0, 1.09)	-0.41 (-3.32, 1.21)	-0.38 (-3.35, 0.78)	-0.21 (-0.51, 0)
**3**	1.59 (1.28, 1.96)*	2.01 (1.14, 2.75)*	0.57 (0, 3.96)	0.19 (0, 2.26)	0.14 (-1.58, 2.27)	-0.1 (-2.13, 1.4)	-0.1 (-0.38, 0)
**4**	1.63 (1.28, 2.05)*	1.77 (1.07, 2.41)*	1.3 (0, 5.97)	0.05 (0, 1)	0.98 (0, 3.55)	-0.13 (-2.23, 1.25)	-0.06 (-0.32, 0.07)
**5**	1.37 (1, 1.75)*	1.67 (1.02, 2.31)*	2.06 (0, 7.24)	0.14 (0, 2.15)	1.96 (0, 4.74)	0.14 (-1.05, 2.29)	-0.05 (-0.3, 0.08)
**6**	1.34 (0.77, 1.9)*	1.51 (0.83, 2.19)*	1.59 (0, 6.4)	0.19 (0, 2.63)	2.08 (0, 4.94)	0.66 (0, 3.69)	-0.06 (-0.3, 0.06)
**7**	-0.12 (-0.69, 0)	-1.56 (-2.52, -0.51)*	1.9 (0, 7.09)	0.12 (0, 1.97)	2.41 (0, 5.13)	0.45 (0, 3.14)	0.07 (-0.04, 0.35)
**8**	-0.9 (-1.32, -0.47)*	-1.89 (-2.65, -1.13)*	1.96 (0, 7.08)	0.32 (0, 3.39)	1.65 (0, 4.27)	1.5 (0, 5.2)	0.1 (0, 0.39)
**9**	-1.01 (-1.44, -0.5)*	-1.88 (-2.59, -1.18)*	1.01 (0, 5.4)	0.13 (0, 1.94)	1.51 (0, 4.09)	1.58 (0, 5.35)	0.12 (0, 0.43)
**10**	-1.28 (-1.55, -1)*	-1.82 (-2.51, -1.01)*	1.32 (0, 6.27)	0.05 (0, 0.65)	1.03 (0, 3.51)	1.3 (0, 5.02)	0.15 (0, 0.46)
**11**	-1.49 (-1.84, -1.17)*	-1.81 (-2.51, -0.88)*	1.13 (0, 5.81)	0.04 (0, 0.45)	1.08 (0, 3.66)	1.16 (0, 4.69)	0.12 (0, 0.41)
**12**	-1.68 (-2.4, -1.07)*	-1.87 (-2.57, -0.96)*	1.06 (-0.3, 6.14)	0.01 (-0.13, 0)	1.14 (0, 3.77)	0.25 (-0.78, 2.56)	0.18 (0, 0.49)
**13**	-1.99 (-3.21, -1.02)*	-1.63 (-2.36, -0.55)*	0.86 (-0.97, 6.13)	-0.14 (-2.19, 0)	0.61 (-0.62, 3.24)	0.18 (-1.07, 2.41)	0.07 (-0.05, 0.33)
**14**	-2.59 (-4.67, -1.1)*	-1.61 (-2.38, -0.34)*	0.68 (-2.72, 9.16)	-1.26 (-6.45, 0)	1.11 (0, 4.49)	0.14 (-1.1, 2.4)	-0.1 (-0.4, 0.01)

^1^Lags refer to the daily lagged covariate

^2^Daily number of reported dengue cases

^3^Daily number of reported sporadic dengue cases (i.e. Isolated cases that have no epidemiological link)

^4^Daily mean absolute humidity

^5^Daily mean temperature

^6^Daily mean maximum temperature

^7^Daily mean minimum temperature

^8^Daily mean rainfall

*denotes statistical significance at the 95% level

Estimates for *R*_*t*,*EpiFilter*_ were aggregated temporally to the mean *R*_*t*,*EpiFilter*_ and percentage of time *R*_*t*,*EpiFilter*_*>* 1.0 to allow us to examine the spatial relationships in effective reproduction numbers themselves and between related confounders. Under the Moran’s I statistic, we found that the mean *R*_*t*,*EpiFilter*_ and percentage of time *R*_*t*,*EpiFilter*_ *>* 1.0 were highly spatially autocorrelated (Table 2).

The spatial distribution of the spatial covariates are presented in S1 Fig. Among the spatial covariates, impervious surfaces, vegetation with structure dominated by human management (without tree canopy) and premise type were found to be spatially associated with the mean *R*_*t*,*EpiFilter*_ estimates. A 1% increase in impervious surfaces and vegetation with structure dominated by human management were associated with a 0.04 (Table 4: 95% CI: 0.03–0.05) and 0.03 (Table 4: 95% CI: 0.01–0.04) increase in the mean *R*_*t*,*EpiFilter*_ respectively. Among the premise type, landed homes were associated with higher mean *R*_*t*,*EpiFilter*_ (Table 4: *β* = 0.01, 95% CI: 0.01–0.01). When using the percentage of time *R*_*t*,*EpiFilter*_ *>* 1.0 as the dependent variable, the landed homes were associated with an increased percentage while the vegetation with structure dominated by human management (with tree canopy) was associated with a reduction in percentage (Table 4: *β* = -0.61%, 95% CI: -1.10%–-0.11%).

**Table 4:** Regression coefficients and associated 95% confidence interval for spatial analysis, where the dependent variable refers to mean *R*_*t*,*EpiFilter*_ and percentage *R*_*t*,*EpiFilter*_ > 1.0 from 2010 to 2020.

Variable	Mean *R*_*t*,*EpiFilter*_	Percentage *R*_*t*,*EpiFilter*_*>*1.0
Freshwater	-0.00 (-0.14, 0.01)	0.04 (-3.91, 4.72)
Non-vegetated pervious surfaces	-0.01 (-0.14, 0.01)	-0.26 (-4.47, 3.98)
Impervious surfaces	0.04 (0.03, 0.05)*	0.32 (-3.74, 4.38)
Vegetation^1^	-0.03 (-0.15, 0.10)	-0.61 (-1.10, -0.11)*
Vegetation^2^	0.03 (0.01, 0.04)*	-0.23 (-3.87, 4.33)
Vegetation^3^	-0.15 (-0.14, 0.01)	-0.10 (-4.32, 4.12)
Vegetation^4^	-0.02 (-0.02, 0.01)	-0.31 (-5.11, 4.49)
Population	0.00 (0.00, 0.00)	0.00 (0.00, 0.00)
Premise Type	0.01 (0.01, 0.01)*	0.20 (0.11, 0.29)*

^1^with structure dominated by human management (with tree canopy)

^2^with structure dominated by human management (without tree canopy)

^3^with limited human management (with tree canopy)

^4^with limited human management (without tree canopy)

^5^public high-rise apartments as referent

*denotes statistical significance at 95% level

## 4. Discussion

Quantifying disease transmissibility is crucial for understanding the epidemiology of infectious diseases, and it helps in the design of effective control measures to facilitate outbreak preparedness. Crucially, assessment of disease transmissibility allows policy makers to be aware of the disease situation in real-time. Although the concept of effective reproduction numbers is well-established, its application as a public health surveillance index had only experienced increased popularity in recent times. Due to the COVID-19 pandemic, it is now viewed as a convenient and useful index for surveillance of infectious diseases [18–19,38]. Many studies have used effective reproduction numbers to monitor near real-time changes in the transmission of respiratory pathogens such as SARS-CoV-1 [19], SARS-CoV-2 [18] and seasonal influenza [39]. They have proven useful in providing important insights into the temporal changes in transmission as well as evaluating in real-time the efficacy of control measures [40]. However, the effective reproduction number was rarely used for dengue, primarily due to concerns with dengue’s spatially and climatically influenced generation interval. Therefore, our study adds to the existing literature on dengue surveillance by demonstrating the utility of using effective reproduction numbers as a real-time dengue surveillance tool for detecting outbreaks and guiding intervention in Singapore. We quantified effective reproduction numbers at both national and local levels, and examined the spatial and temporal variation of the estimates in relation to a wide range of environmental and anthropogenic factors.

We found that the lagged reported dengue case counts and sporadic case counts were associated with dengue transmissibility at the national level. This is to be expected given that dengue case counts were used to calibrate the statistical models for estimating effective reproduction numbers. The difference in the direction of association at the different lags is likely attributable to the generation interval distribution, in which the average infectiousness profile of dengue first increases and then decreases with time. More importantly, although dengue cases were known to be affected by the weather [41–44], we found no residual relationship between climate factors and dengue transmissibility once disease case counts were controlled for.

Our analysis revealed that the eastern regions of Singapore experience higher dengue transmission intensity. This corresponds with historical spatial trends for dengue indicating that the eastern regions had comparatively higher levels of reported dengue cases [45]. We examined and identified relevant spatial characteristics that influenced dengue transmission intensity at the local level. First, the proportion of impervious surfaces was positively associated with dengue transmission intensity. This is not surprising since impervious surfaces are a proxy indicator for urbanization, which provides favourable breeding habitats for *Ae*. *aegypti* mosquitoes that thrive in urban environments [46–47]. Next, the transmission intensity of dengue was positively associated with increased proportion of vegetation with structure dominated by human management (without tree canopy). This is likely due to the increased availability of water in leaf litter, soil surface, pots and in the discarded receptacles hidden in the foliage or shrub, which supports mosquito breeding [47–48]. In contrast, vegetation with structure dominated by human management (with tree canopy) was associated with lower dengue transmission intensity. Although *Ae*. *albopictus* is native and ubiquitous throughout Singapore, the status of *Ae*. *aegypti* and *Ae*. *albopictus* as dengue vectors in Singapore mirrors the global situation, in which *Ae*. *aegypti* is the primary vector while *Ae*. *albopictus* is a less efficient vector. Furthermore, *Ae*. *aegypti* prefer highly urbanized areas so they are unlikely to be found in forested areas [49–50]. The intensity of transmission was also found to be higher in landed houses than in public high-rise apartments. This is consistent with previous studies conducted in Singapore, which showed that landed properties generally have higher incidences of dengue [51–52]. The topography of landed residential homes is much more favorable to mosquito breeding than high rise residences. The larger surface area and greater variety of structures and receptacle types within landed residential home compounds make them conducive for harbouring mosquito breeding habitats [48].

To the best of our knowledge, our study is the first to apply effective reproduction numbers as a surveillance index for dengue and to estimate the effective reproduction number of dengue on a fine spatial scale. In addition, we nested generation interval uncertainty that included both the vector and host incubation periods in our estimation of effective reproduction numbers. We showed that both methods, EpiEstim and EpiFilter, are comparable in terms of model performance and fit the dengue case data well at the national level. The EpiEstim method, however, had poorer performances under three model assessment measures and converged to prior assumptions under low case counts. Therefore, the EpiFilter method provided more reliable estimates of effective reproduction numbers in this scenario. We also showed that dengue outbreaks were preceded by sustained periods of high transmissibility. This demonstrates that estimates of effective reproduction numbers can detect large rises in dengue case counts, supporting the utility of effective reproduction numbers as a dengue surveillance tool. Real-time monitoring of effective reproduction numbers can assist public health agencies in identifying high transmission risk areas and prioritizing the allocation of control measures.

There are, however, some limitations to our study. Firstly, we assumed that all cases were transmitted locally and that there were no imported cases. Failure to account for imported cases can lead to an overestimation of the effective reproduction number. However, the proportion of imported cases in Singapore is very low, accounting for only less than 4% of total case count. Secondly, the estimates of effective reproduction number obtained under EpiEstim method are sensitive to the size of the sliding window over which the estimates are calculated. Smaller windows produce highly variable estimates with wide credible intervals, whereas longer windows lead to smoothed estimates with narrower credible intervals. Thirdly, the methods for estimating *R*_*t*_ require that the observation rate for dengue cases be stable over time. This hold true in Singapore, where dengue is endemic, with an average of 34.3 dengue cases observed daily, far exceeding the suggested minimum of 12 cases in [14]. In addition, the surface land cover data and the population-based statistics were based on a single year, 2018, and we assumed that these spatial covariates did not vary substantially over time. Next, several studies have shown that *Ae*. *aegypti* abundance is associated with increased transmission [2,45,53–54], however, due to data availability, we were unable to account for the effects of *Ae*. *aegypti* population on dengue transmissibility in our study. Also, we did not account for variation in the transmissibility of each of the four dengue serotypes that are in circulation in Singapore [55], which might depend on other factors such as levels of population immunity and/or cross-immunity to each serotype. However, given that these dynamics are likely long-term trends, they are unlikely to affect estimates as effective reproduction numbers are short-term metrics [56]. Lastly, while we revealed significant spatial autocorrelation in *R*_*t*_ estimates, these were not explicitly incorporated in our estimates, due to the limitations in the utilised functional form of *R*_*t*_. Future work can consider incorporating a spatially recursive estimator for *R*_*t*_, but would require deriving a new estimator from first principles [57], and the development of tools required to calibrate this estimator to actual data.

## 5. Conclusion

This study demonstrates the potential of *R*_*t*_ as a dengue surveillance tool to complement currently available forecasting models. Effective reproduction numbers produced using proposed methods have high accuracy and provide important insights into the temporal change of dengue transmissibility nationally and at the local level. Real-time estimation of the effective reproduction number can assist public health agencies in identifying areas with high dengue transmission risk and facilitating localised outbreak preparedness and response.

## Supporting information

S1 Fig (A) Percentage of Freshwater (B) Percentage of impervious surfaces (C) Percentage of non-vegetated pervious surfaces (D) Population (E) Percentage of vegetation with structure dominated by human management (tree canopy) (F) Percentage of vegetation with structure dominated by human management (without tree canopy) (G) Percentage of vegetation with limited human management (tree canopy) (H) Percentage of vegetation with limited human management (without tree canopy) (I) Premise type. The figure was created with base layer obtained from https://gadm.org/maps.html.
(TIF)

S1 Appendix Daily reported dengue case counts (Top Panel) and estimated effective reproduction numbers under EpiEstim and EpiFilter methods (Bottom Panel) in 24 spatial units.
(PDF)
