# Supplementary figures and images for: Fine-scale estimation of effective reproduction numbers for dengue surveillance
Source: PLoS Comput Biol. 2022 Jan 20;18(1):e1009791. doi: 10.1371/journal.pcbi.1009791 (PMC8836367; doi:10.1371/journal.pcbi.1009791)

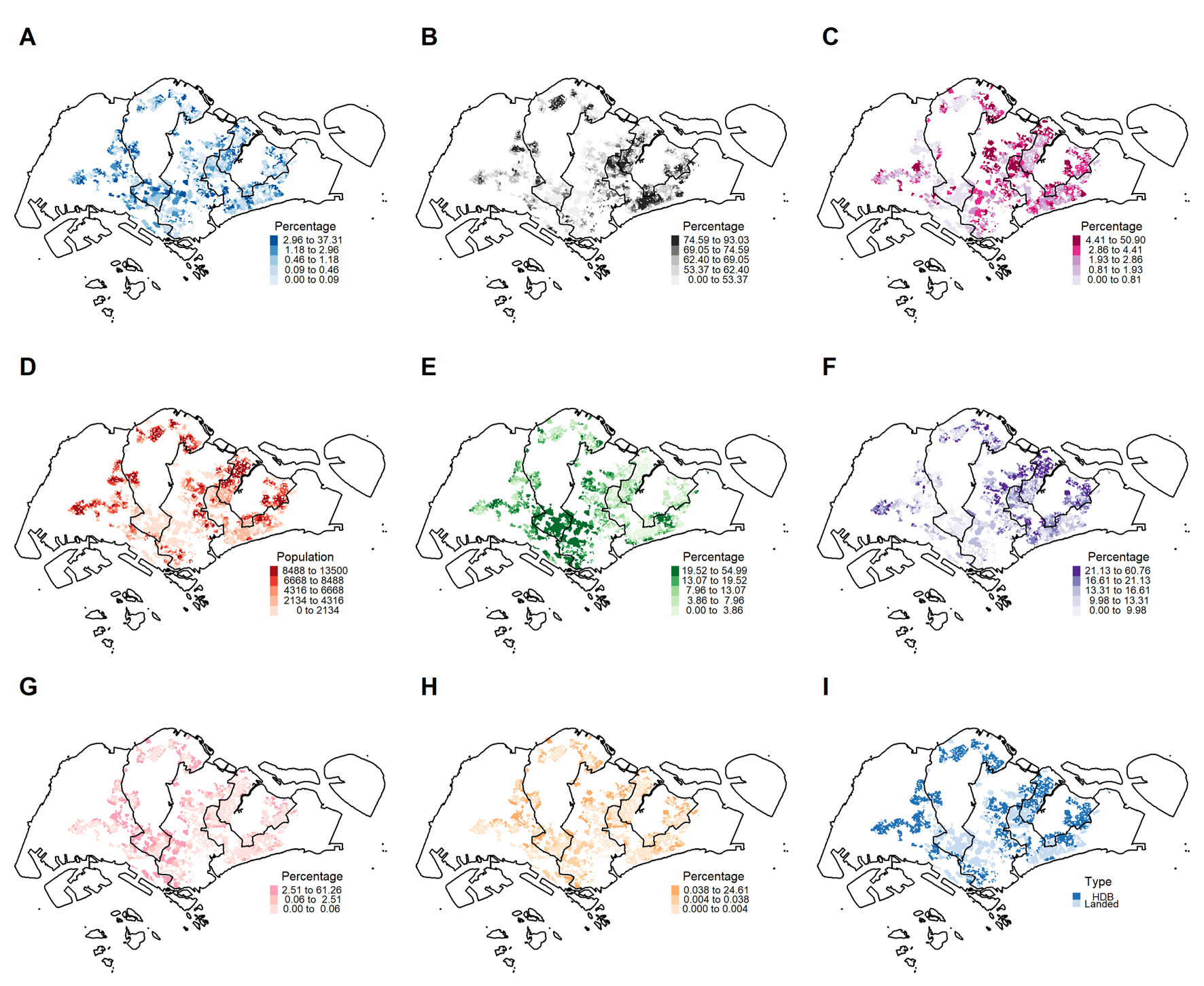

Supplement: S1 Fig — (A) Percentage of Freshwater (B) Percentage of impervious surfaces (C) Percentage of non-vegetated pervious surfaces (D) Population (E) Percentage of vegetation with structure dominated by human management (tree canopy) (F) Percentage of vegetation with structure dominated by human management (without tree canopy) (G) Percentage of vegetation with limited human management (tree canopy) (H) Percentage of vegetation with limited human management (without tree canopy) (I) Premise type. The figure was created with base layer obtained from https://gadm.org/maps.html. (TIF) [file pcbi.1009791.s001.tif]
