# Supplementary material for: Fine-scale estimation of effective reproduction numbers for dengue surveillance
Source: PLoS Comput Biol. 2022 Jan 20;18(1):e1009791. doi: 10.1371/journal.pcbi.1009791 (PMC8836367; doi:10.1371/journal.pcbi.1009791)
Supplement: S1 Appendix — Daily reported dengue case counts (Top Panel) and estimated effective reproduction numbers under EpiEstim and EpiFilter methods (Bottom Panel) in 24 spatial units. (PDF) [file pcbi.1009791.s002.pdf]

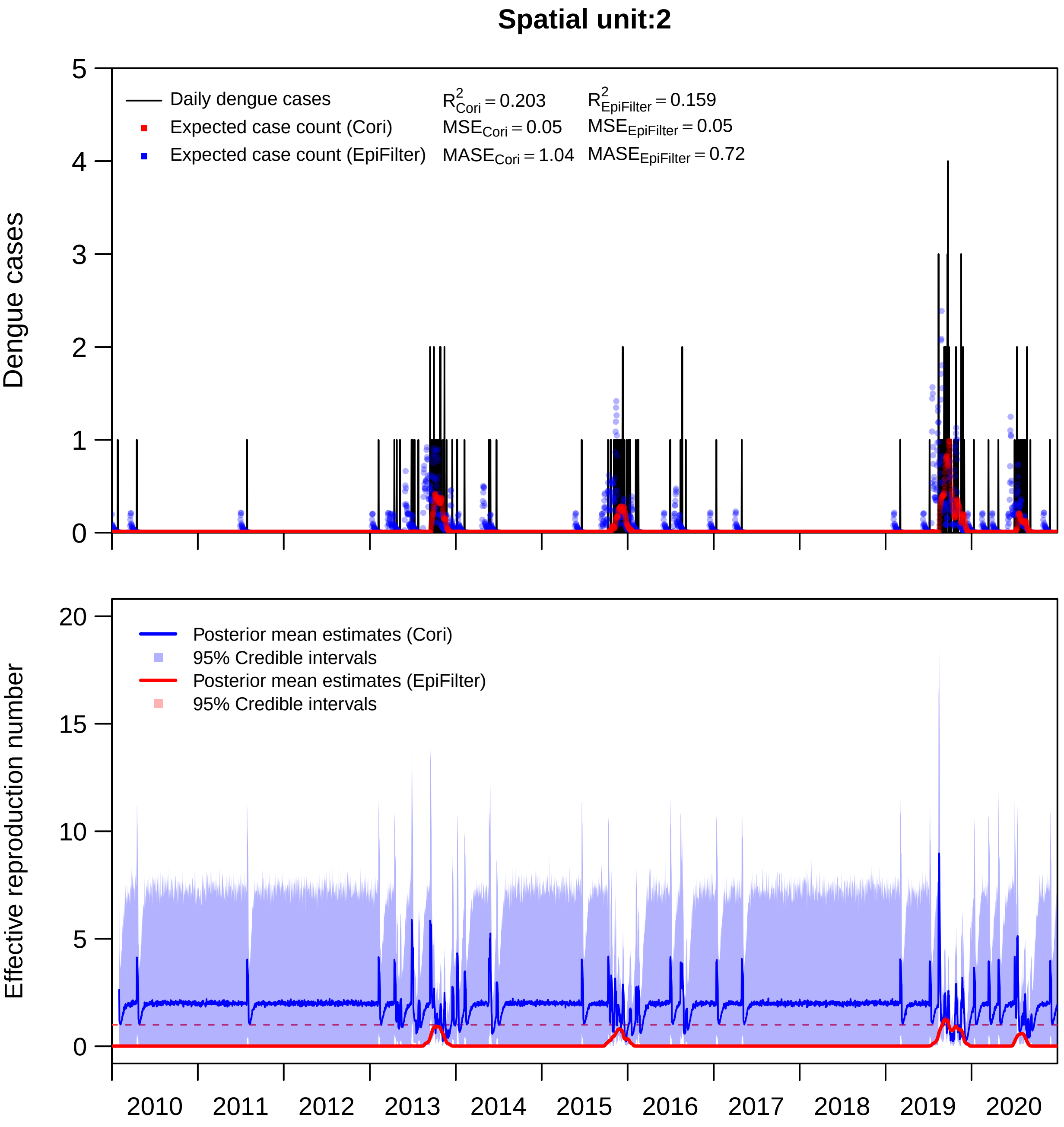
Spatial unit:2
Dengue cases
Daily dengue cases
Expected case count (Cori)
Expected case count (EpiFilter)
$R^2_{Cori} = 0.203$
$MSE_{Cori} = 0.05$
$MASE_{Cori} = 1.04$
$R^2_{EpiFilter} = 0.159$
$MSE_{EpiFilter} = 0.05$
$MASE_{EpiFilter} = 0.72$
0
1
2
3
4
5
Effective reproduction number
Posterior mean estimates (Cori)
95% Credible intervals
Posterior mean estimates (EpiFilter)
95% Credible intervals
0
5
10
15
20
2010
2011
2012
2013
2014
2015
2016
2017
2018
2019
2020

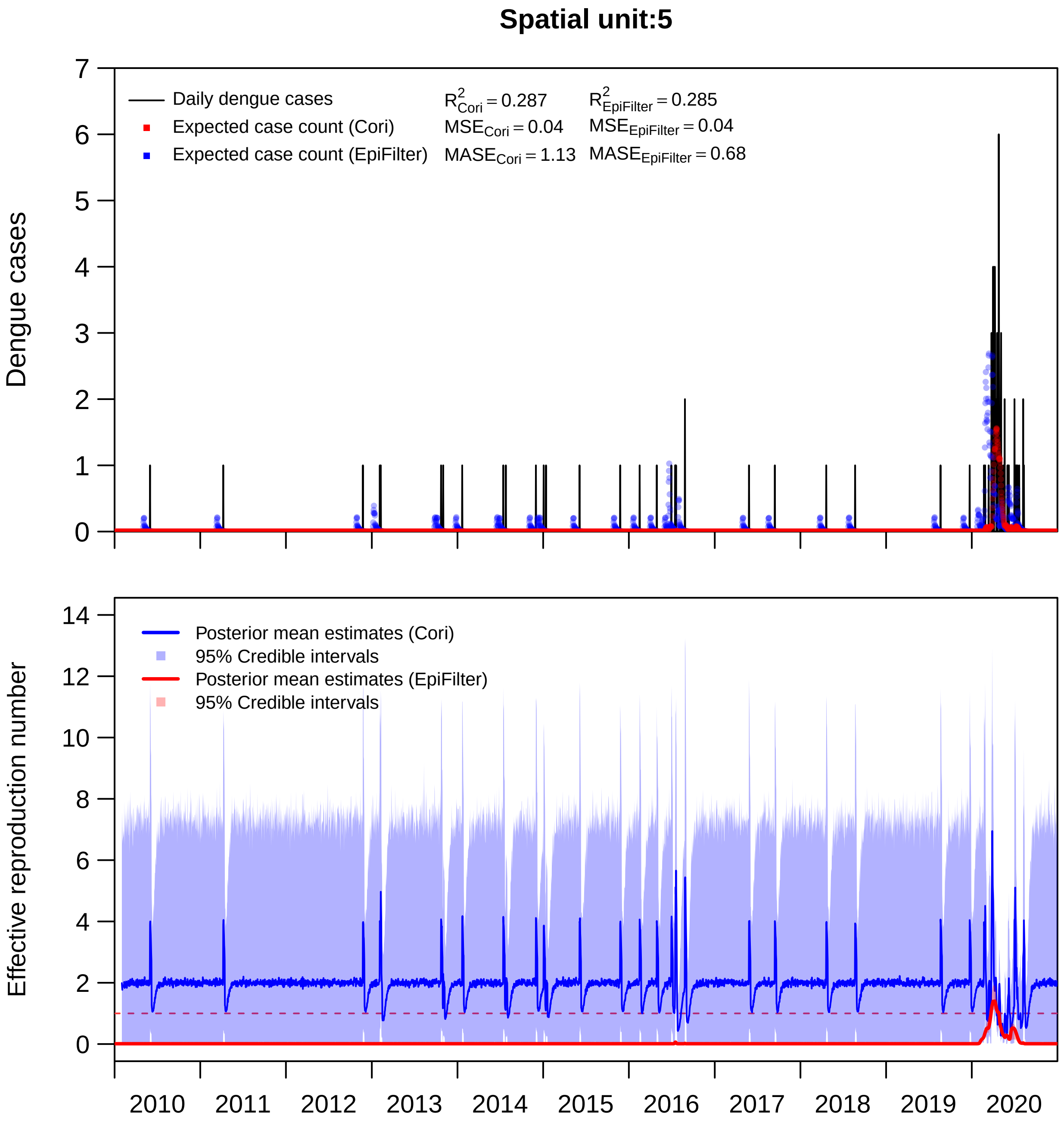
Spatial unit:5
Daily dengue cases
Expected case count (Cori)
Expected case count (EpiFilter)
$R^2_{Cori} = 0.287$
$MSE_{Cori} = 0.04$
$MASE_{Cori} = 1.13$
$R^2_{EpiFilter} = 0.285$
$MSE_{EpiFilter} = 0.04$
$MASE_{EpiFilter} = 0.68$
Dengue cases
Posterior mean estimates (Cori)
95% Credible intervals
Posterior mean estimates (EpiFilter)
95% Credible intervals
Effective reproduction number
2010
2011
2012
2013
2014
2015
2016
2017
2018
2019
2020

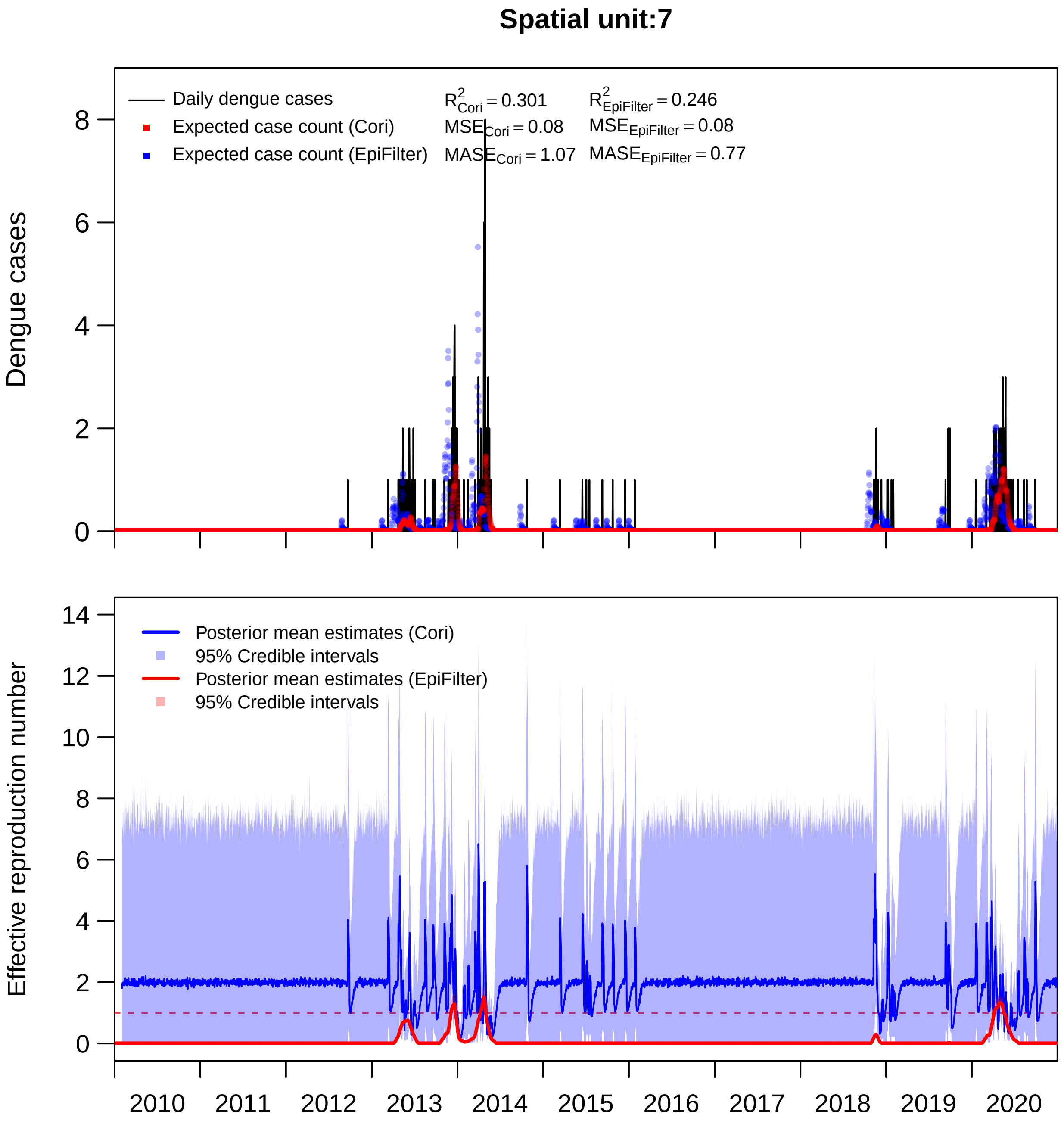

Spatial unit:7
Dengue cases
Daily dengue cases
Expected case count (Cori)
Expected case count (EpiFilter)
$R^2_{Cori} = 0.301$
$MSE_{Cori} = 0.08$
$MASE_{Cori} = 1.07$
$R^2_{EpiFilter} = 0.246$
$MSE_{EpiFilter} = 0.08$
$MASE_{EpiFilter} = 0.77$
0
2
4
6
8
Effective reproduction number
Posterior mean estimates (Cori)
95% Credible intervals
Posterior mean estimates (EpiFilter)
95% Credible intervals
0
2
4
6
8
10
12
14
2010
2011
2012
2013
2014
2015
2016
2017
2018
2019
2020

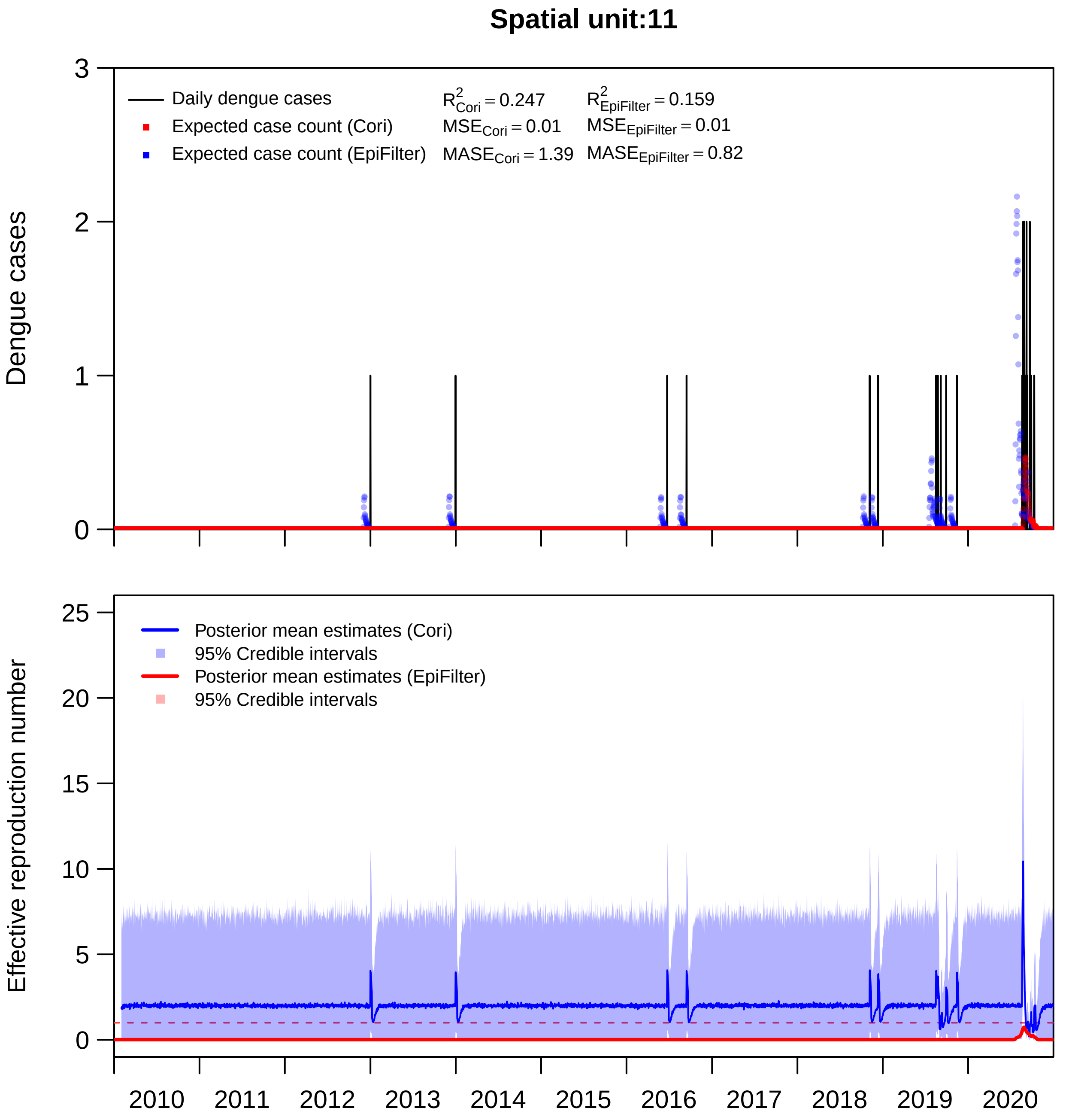

Spatial unit:11
Dengue cases
Daily dengue cases
Expected case count (Cori)
Expected case count (EpiFilter)
$R^2_{Cori} = 0.247$
$MSE_{Cori} = 0.01$
$MASE_{Cori} = 1.39$
$R^2_{EpiFilter} = 0.159$
$MSE_{EpiFilter} = 0.01$
$MASE_{EpiFilter} = 0.82$
0
1
2
3
Effective reproduction number
Posterior mean estimates (Cori)
95% Credible intervals
Posterior mean estimates (EpiFilter)
95% Credible intervals
0
5
10
15
20
25
2010
2011
2012
2013
2014
2015
2016
2017
2018
2019
2020

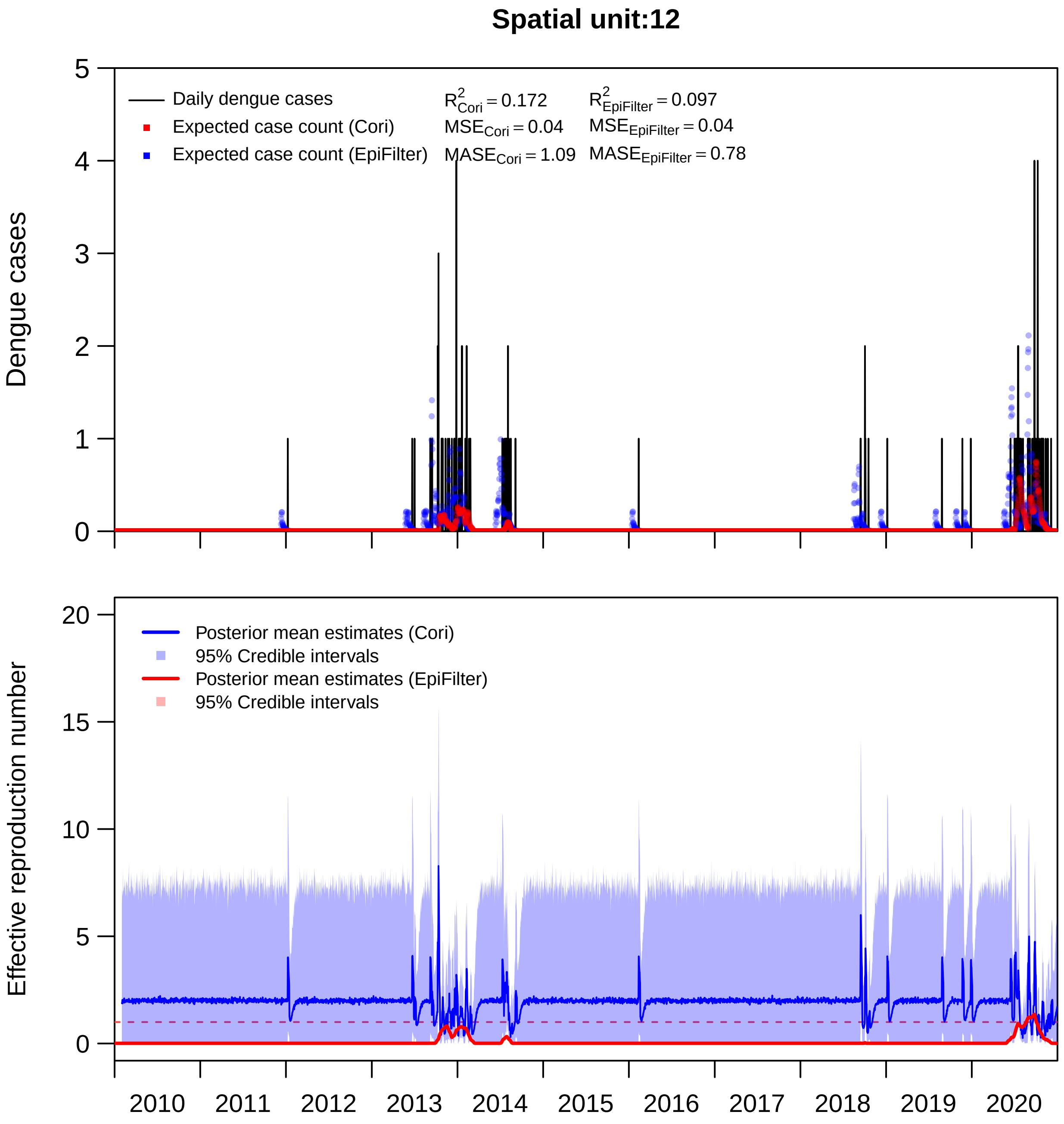

Spatial unit:12
Dengue cases
Daily dengue cases
Expected case count (Cori)
Expected case count (EpiFilter)
R²Cori = 0.172
MSECori = 0.04
MASECori = 1.09
R²EpiFilter = 0.097
MSEEpiFilter = 0.04
MASEEpiFilter = 0.78
0
1
2
3
4
5
Effective reproduction number
Posterior mean estimates (Cori)
95% Credible intervals
Posterior mean estimates (EpiFilter)
95% Credible intervals
0
5
10
15
20
2010
2011
2012
2013
2014
2015
2016
2017
2018
2019
2020

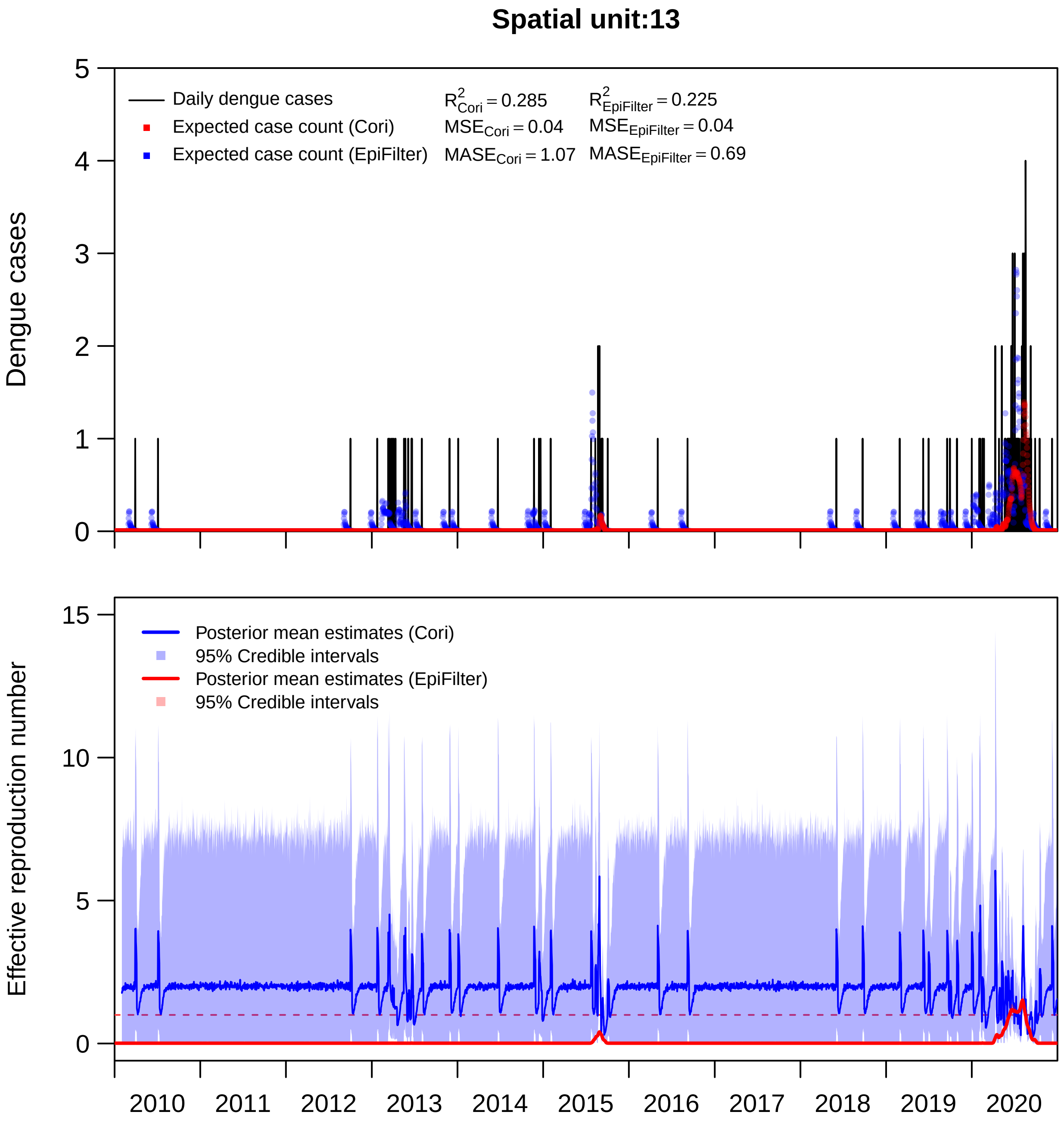

Spatial unit:13
Dengue cases
Daily dengue cases
Expected case count (Cori)
Expected case count (EpiFilter)
$R^2_{Cori} = 0.285$
$MSE_{Cori} = 0.04$
$MASE_{Cori} = 1.07$
$R^2_{EpiFilter} = 0.225$
$MSE_{EpiFilter} = 0.04$
$MASE_{EpiFilter} = 0.69$
0
1
2
3
4
5
Effective reproduction number
Posterior mean estimates (Cori)
95% Credible intervals
Posterior mean estimates (EpiFilter)
95% Credible intervals
0
5
10
15
2010
2011
2012
2013
2014
2015
2016
2017
2018
2019
2020

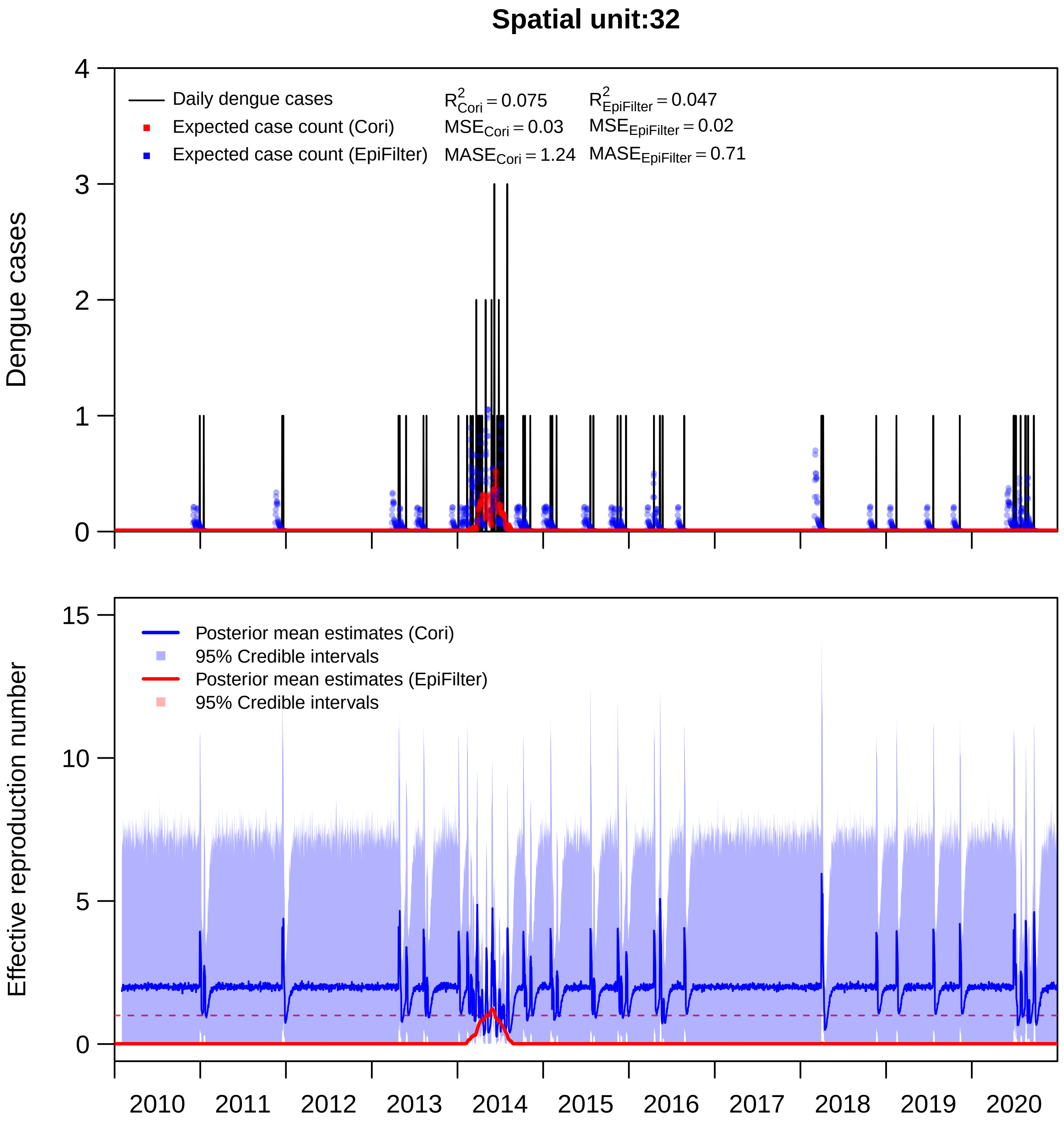

Spatial unit:32
Dengue cases
Daily dengue cases
Expected case count (Cori)
Expected case count (EpiFilter)
$R^2_{Cori} = 0.075$
$MSE_{Cori} = 0.03$
$MASE_{Cori} = 1.24$
$R^2_{EpiFilter} = 0.047$
$MSE_{EpiFilter} = 0.02$
$MASE_{EpiFilter} = 0.71$
0
1
2
3
4
Effective reproduction number
Posterior mean estimates (Cori)
95% Credible intervals
Posterior mean estimates (EpiFilter)
95% Credible intervals
0
5
10
15
2010
2011
2012
2013
2014
2015
2016
2017
2018
2019
2020

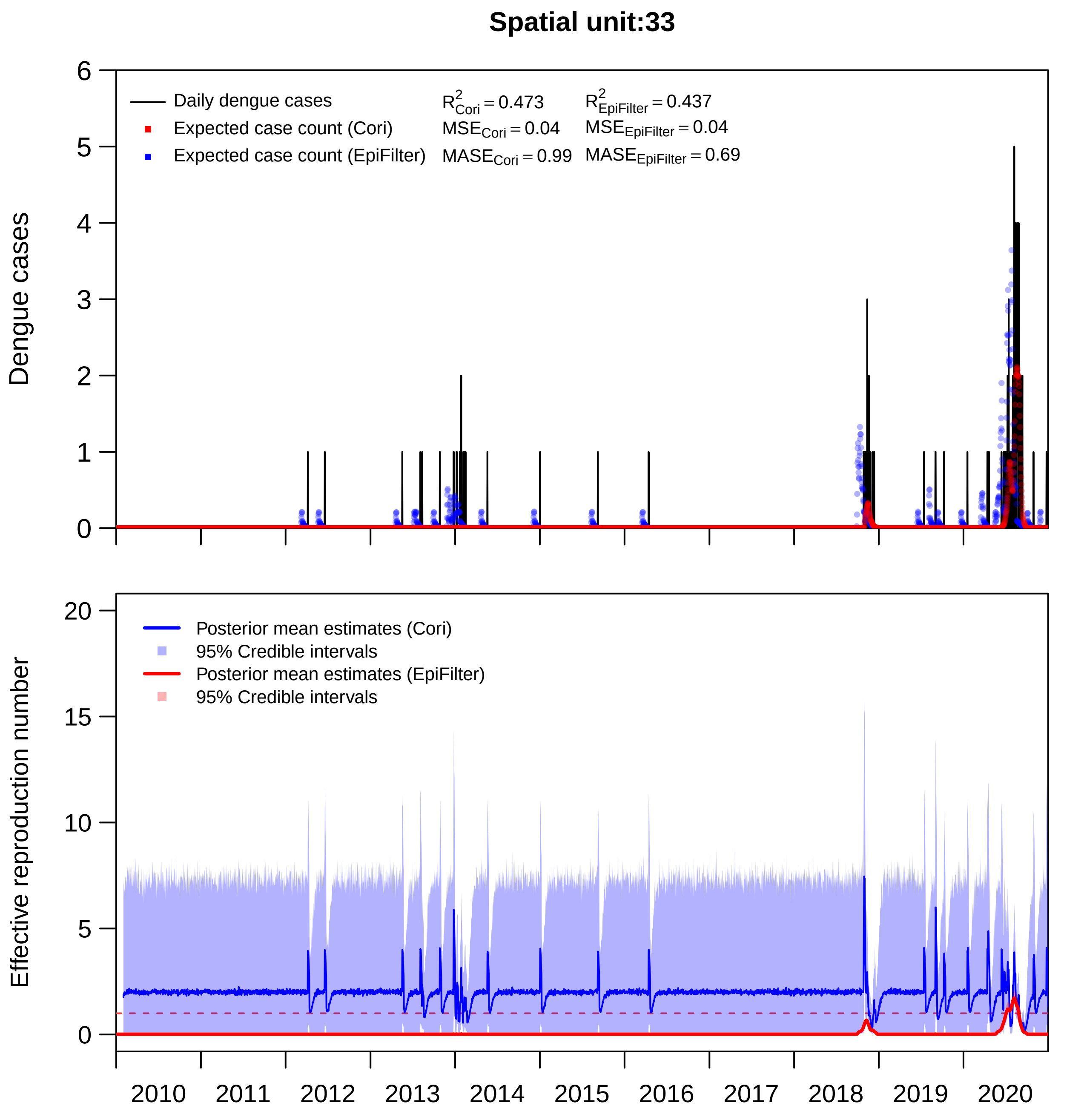

Spatial unit:33
Dengue cases
Daily dengue cases
Expected case count (Cori)
Expected case count (EpiFilter)
$R^2_{Cori} = 0.473$
$MSE_{Cori} = 0.04$
$MASE_{Cori} = 0.99$
$R^2_{EpiFilter} = 0.437$
$MSE_{EpiFilter} = 0.04$
$MASE_{EpiFilter} = 0.69$
0
1
2
3
4
5
6
Effective reproduction number
Posterior mean estimates (Cori)
95% Credible intervals
Posterior mean estimates (EpiFilter)
95% Credible intervals
0
5
10
15
20
2010
2011
2012
2013
2014
2015
2016
2017
2018
2019
2020

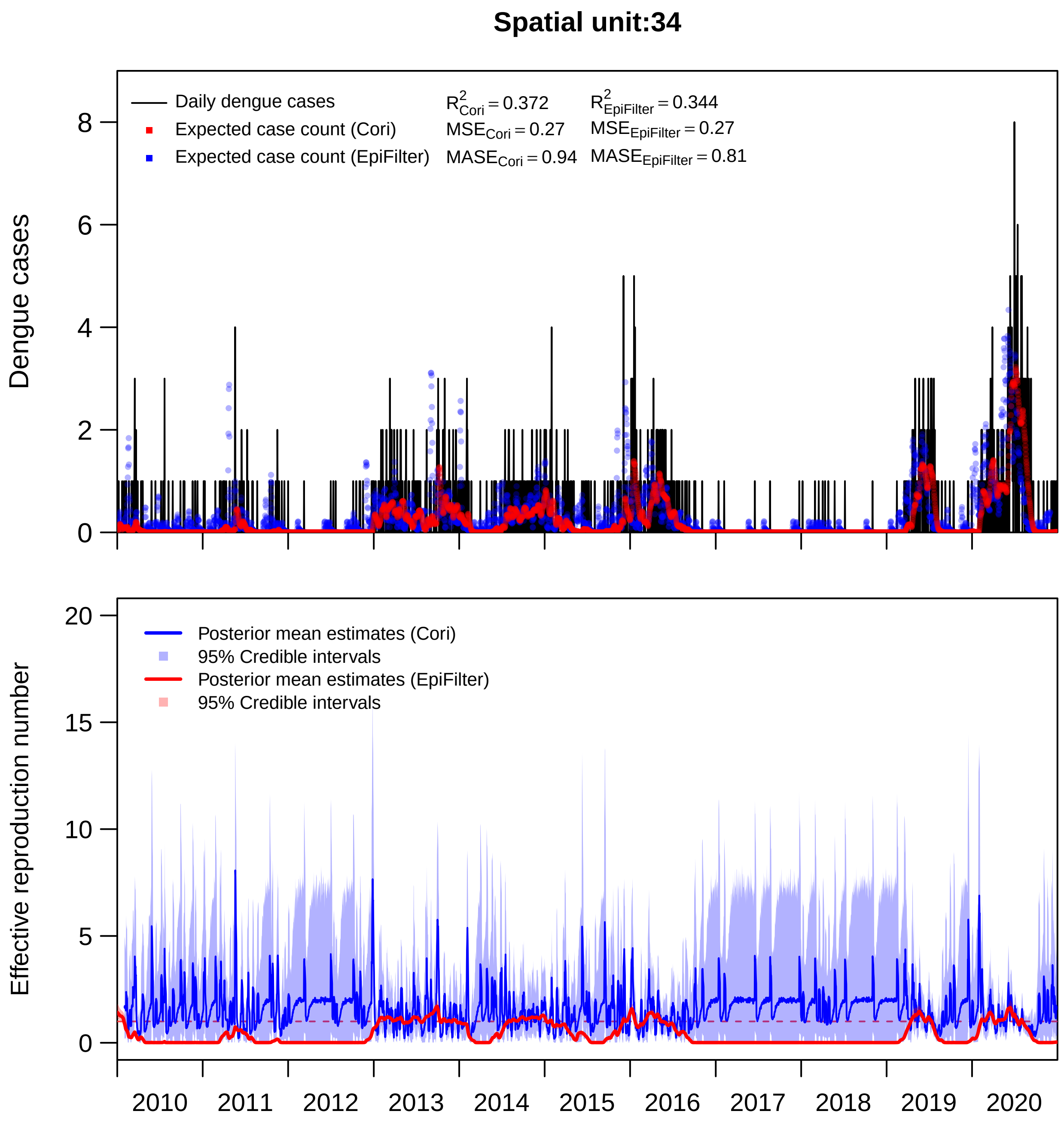

Spatial unit:34
Dengue cases
Daily dengue cases
Expected case count (Cori)
Expected case count (EpiFilter)
$R^2_{Cori} = 0.372$
$MSE_{Cori} = 0.27$
$MASE_{Cori} = 0.94$
$R^2_{EpiFilter} = 0.344$
$MSE_{EpiFilter} = 0.27$
$MASE_{EpiFilter} = 0.81$
0
2
4
6
8
Effective reproduction number
Posterior mean estimates (Cori)
95% Credible intervals
Posterior mean estimates (EpiFilter)
95% Credible intervals
0
5
10
15
20
2010
2011
2012
2013
2014
2015
2016
2017
2018
2019
2020

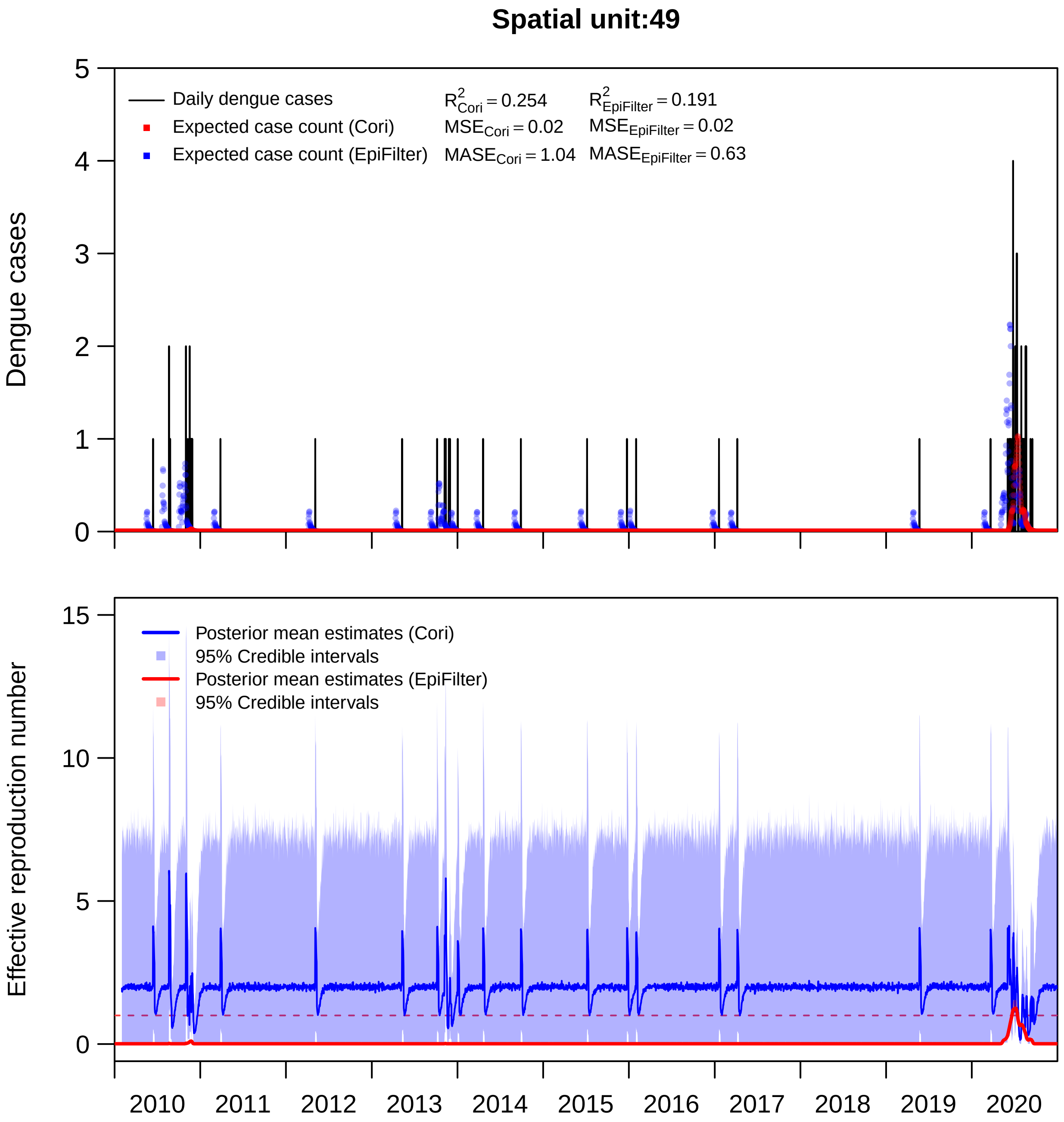

Spatial unit:49
Dengue cases
Daily dengue cases
Expected case count (Cori)
Expected case count (EpiFilter)
$R^2_{Cori} = 0.254$
$MSE_{Cori} = 0.02$
$MASE_{Cori} = 1.04$
$R^2_{EpiFilter} = 0.191$
$MSE_{EpiFilter} = 0.02$
$MASE_{EpiFilter} = 0.63$
0
1
2
3
4
5
Effective reproduction number
Posterior mean estimates (Cori)
95% Credible intervals
Posterior mean estimates (EpiFilter)
95% Credible intervals
0
5
10
15
2010
2011
2012
2013
2014
2015
2016
2017
2018
2019
2020

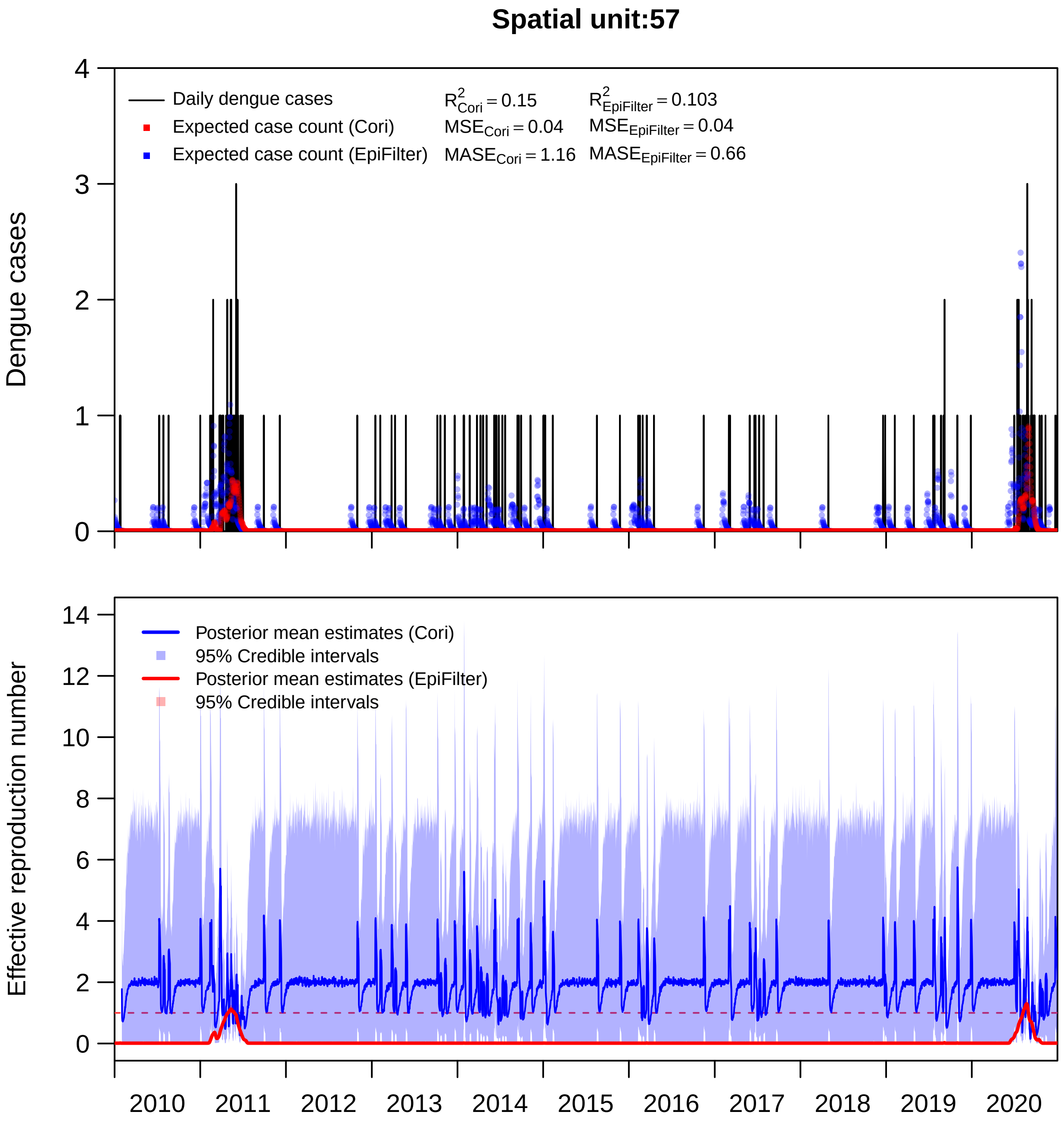

Spatial unit:57
Dengue cases
Daily dengue cases
Expected case count (Cori)
Expected case count (EpiFilter)
$R^2_{Cori} = 0.15$
$MSE_{Cori} = 0.04$
$MASE_{Cori} = 1.16$
$R^2_{EpiFilter} = 0.103$
$MSE_{EpiFilter} = 0.04$
$MASE_{EpiFilter} = 0.66$
Effective reproduction number
Posterior mean estimates (Cori)
95% Credible intervals
Posterior mean estimates (EpiFilter)
95% Credible intervals
2010
2011
2012
2013
2014
2015
2016
2017
2018
2019
2020

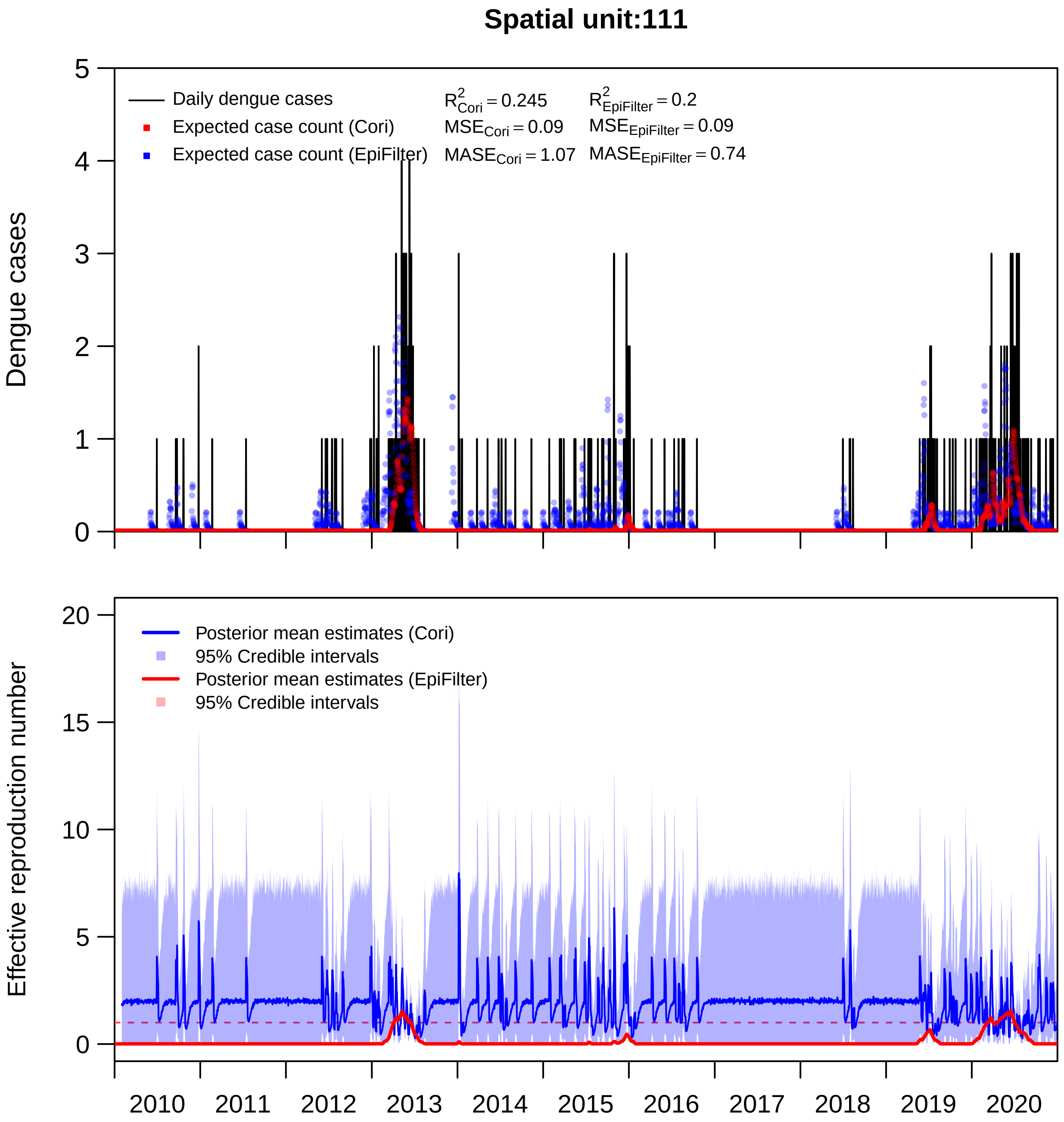

Spatial unit:111
Dengue cases
Daily dengue cases
Expected case count (Cori)
Expected case count (EpiFilter)
$R^2_{Cori} = 0.245$
$MSE_{Cori} = 0.09$
$MASE_{Cori} = 1.07$
$R^2_{EpiFilter} = 0.2$
$MSE_{EpiFilter} = 0.09$
$MASE_{EpiFilter} = 0.74$
Effective reproduction number
Posterior mean estimates (Cori)
95% Credible intervals
Posterior mean estimates (EpiFilter)
95% Credible intervals
2010 2011 2012 2013 2014 2015 2016 2017 2018 2019 2020

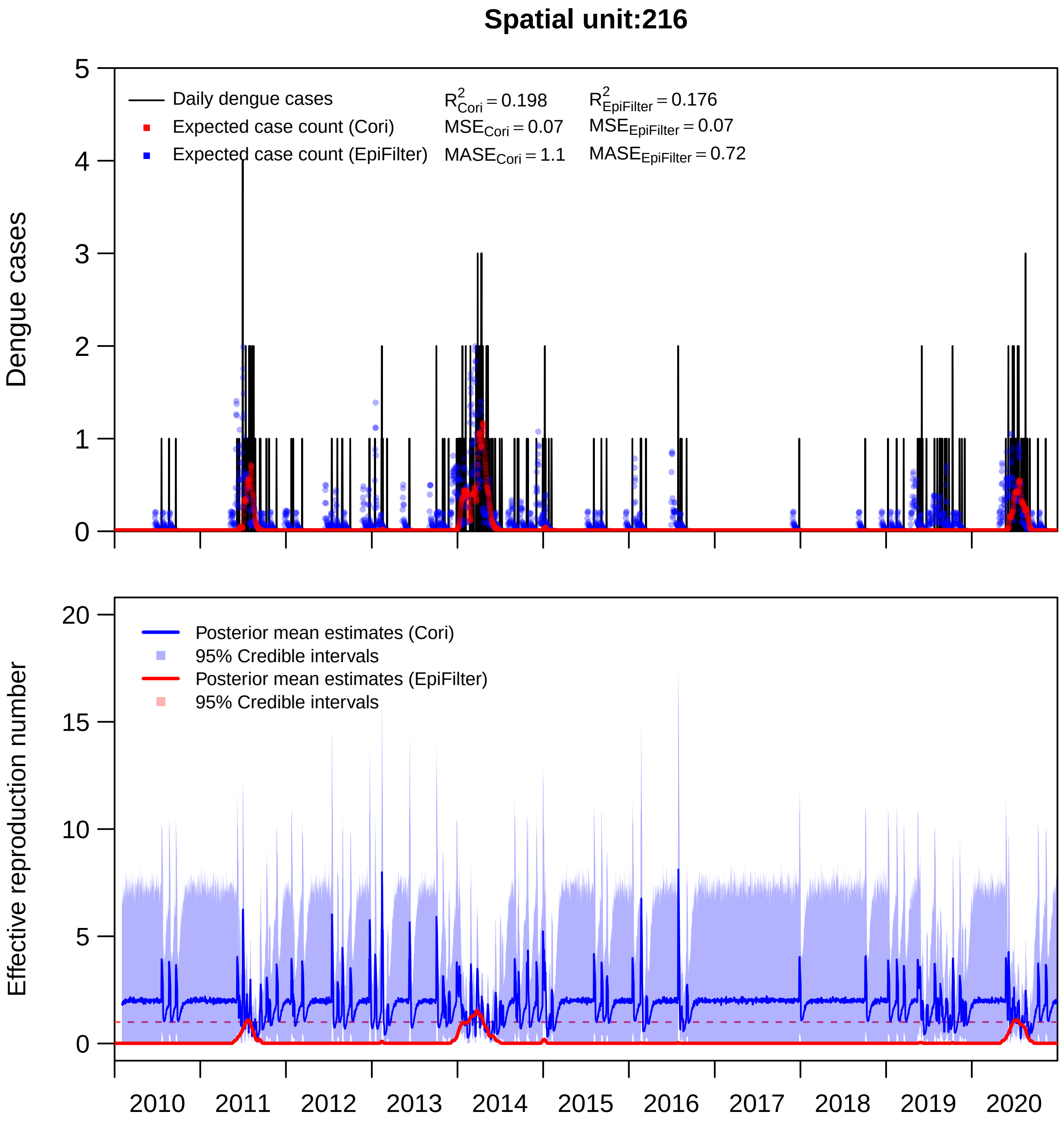

Spatial unit:216
Dengue cases
Daily dengue cases
Expected case count (Cori)
Expected case count (EpiFilter)
$R^2_{Cori} = 0.198$
$MSE_{Cori} = 0.07$
$MASE_{Cori} = 1.1$
$R^2_{EpiFilter} = 0.176$
$MSE_{EpiFilter} = 0.07$
$MASE_{EpiFilter} = 0.72$
Effective reproduction number
Posterior mean estimates (Cori)
95% Credible intervals
Posterior mean estimates (EpiFilter)
95% Credible intervals
2010
2011
2012
2013
2014
2015
2016
2017
2018
2019
2020

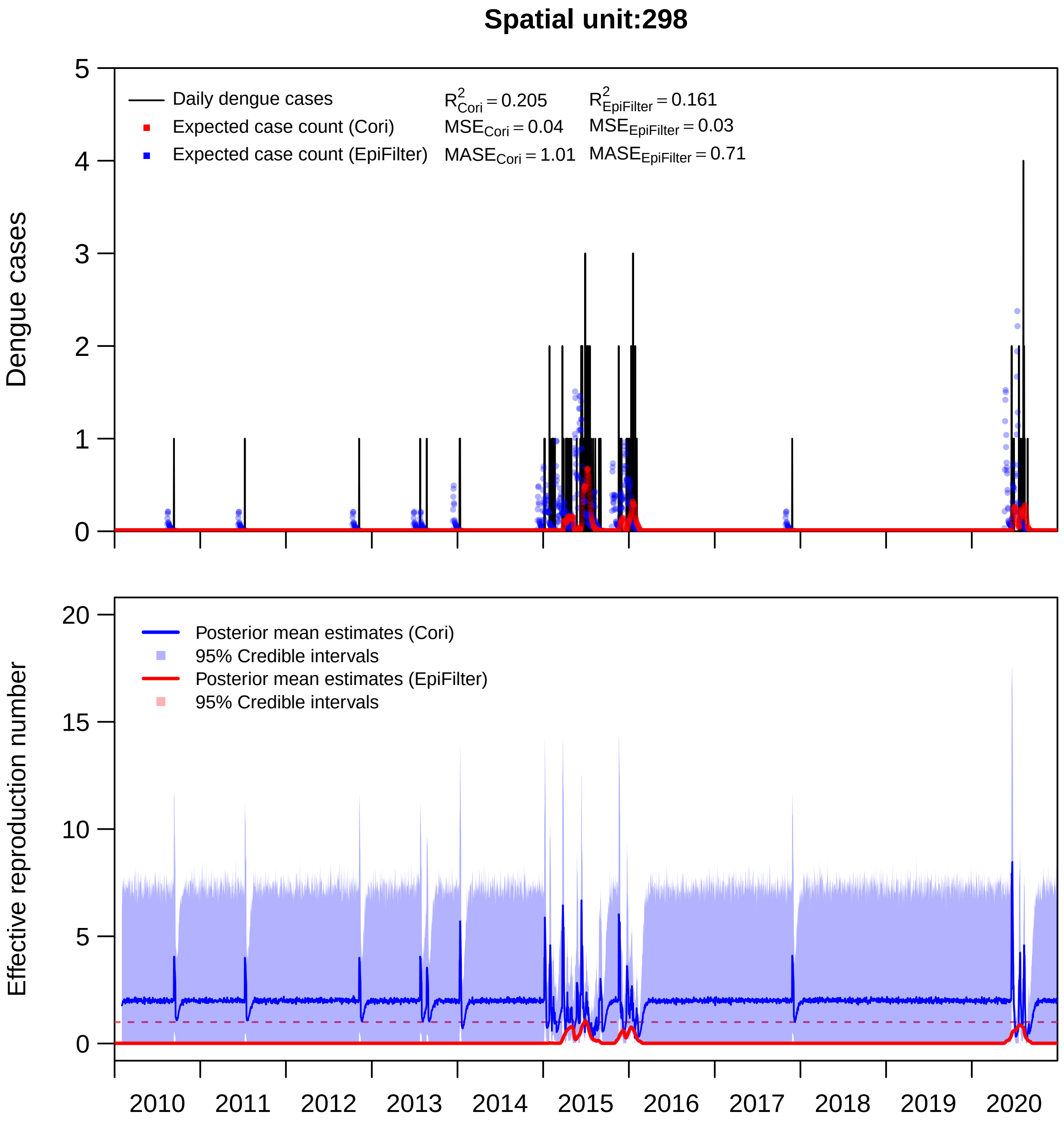

Spatial unit:298
Dengue cases
Daily dengue cases
Expected case count (Cori)
Expected case count (EpiFilter)
$R^2_{Cori} = 0.205$
$MSE_{Cori} = 0.04$
$MASE_{Cori} = 1.01$
$R^2_{EpiFilter} = 0.161$
$MSE_{EpiFilter} = 0.03$
$MASE_{EpiFilter} = 0.71$
0
1
2
3
4
5
Effective reproduction number
Posterior mean estimates (Cori)
95% Credible intervals
Posterior mean estimates (EpiFilter)
95% Credible intervals
0
5
10
15
20
2010
2011
2012
2013
2014
2015
2016
2017
2018
2019
2020

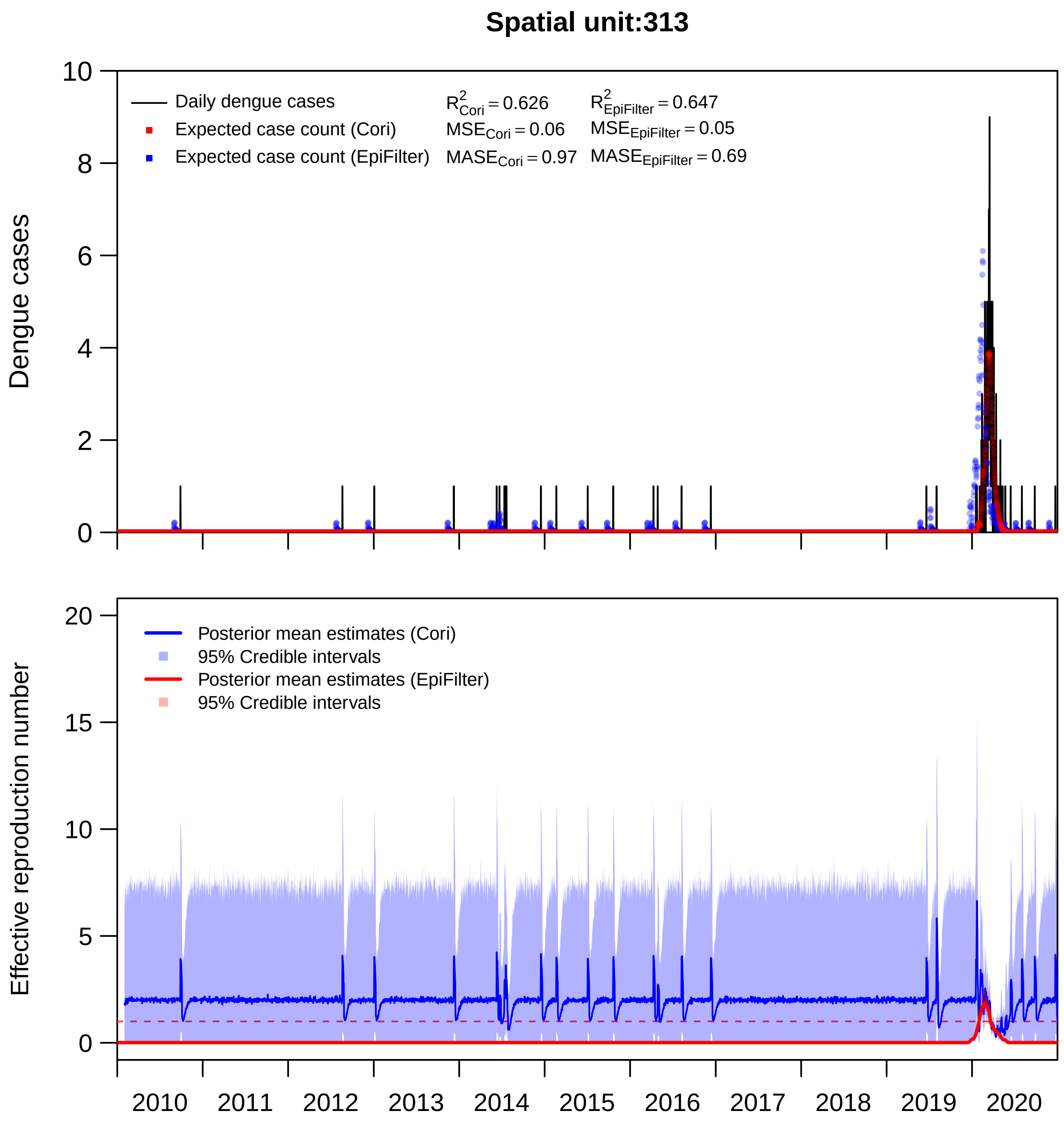
Spatial unit:313
Dengue cases
Daily dengue cases
Expected case count (Cori)
Expected case count (EpiFilter)
$R^2_{Cori} = 0.626$
$MSE_{Cori} = 0.06$
$MASE_{Cori} = 0.97$
$R^2_{EpiFilter} = 0.647$
$MSE_{EpiFilter} = 0.05$
$MASE_{EpiFilter} = 0.69$
Effective reproduction number
Posterior mean estimates (Cori)
95% Credible intervals
Posterior mean estimates (EpiFilter)
95% Credible intervals
2010
2011
2012
2013
2014
2015
2016
2017
2018
2019
2020

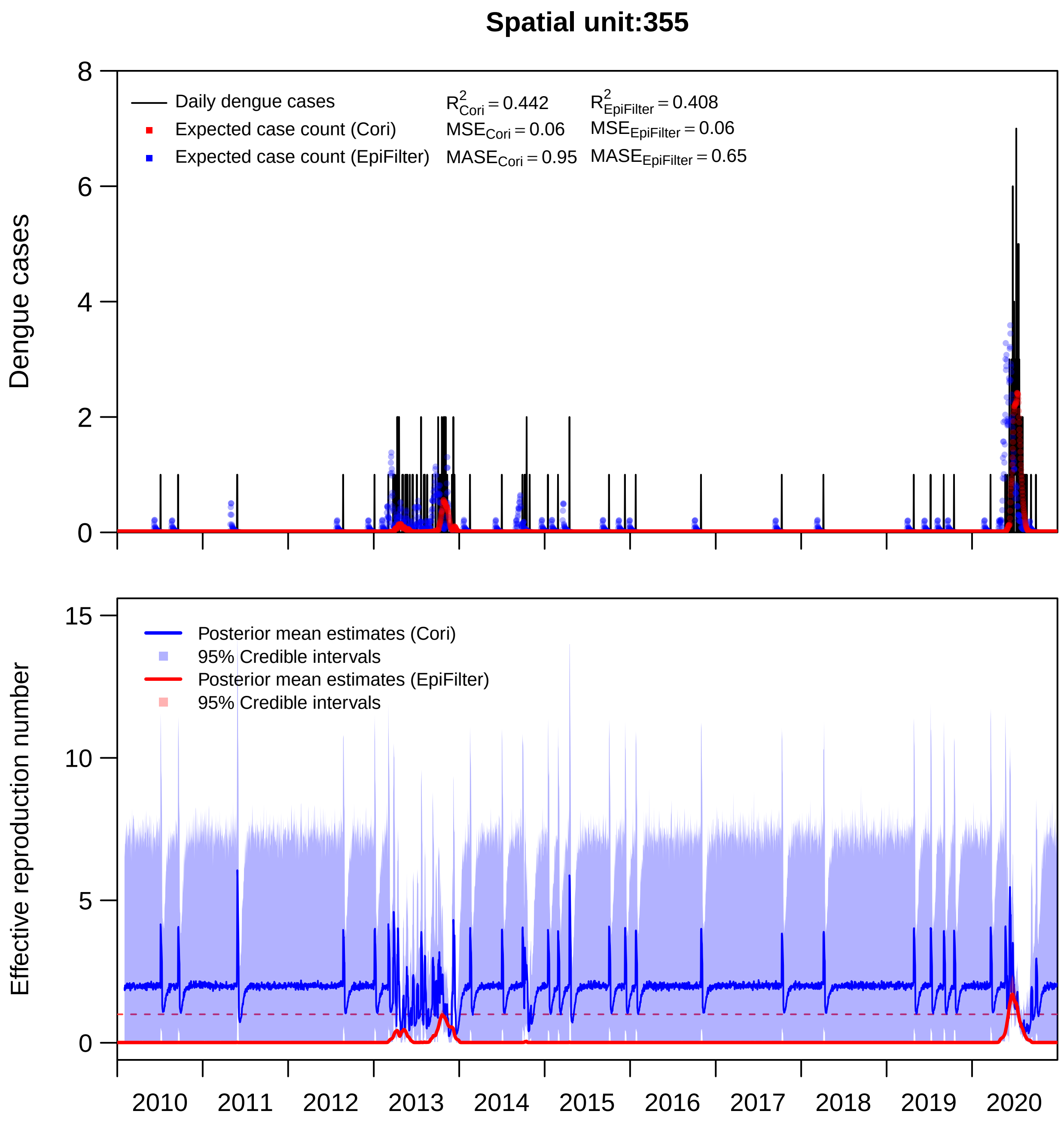
Spatial unit:355
Dengue cases
Daily dengue cases
Expected case count (Cori)
Expected case count (EpiFilter)
$R^2_{Cori} = 0.442$
$MSE_{Cori} = 0.06$
$MASE_{Cori} = 0.95$
$R^2_{EpiFilter} = 0.408$
$MSE_{EpiFilter} = 0.06$
$MASE_{EpiFilter} = 0.65$
Effective reproduction number
Posterior mean estimates (Cori)
95% Credible intervals
Posterior mean estimates (EpiFilter)
95% Credible intervals
2010
2011
2012
2013
2014
2015
2016
2017
2018
2019
2020

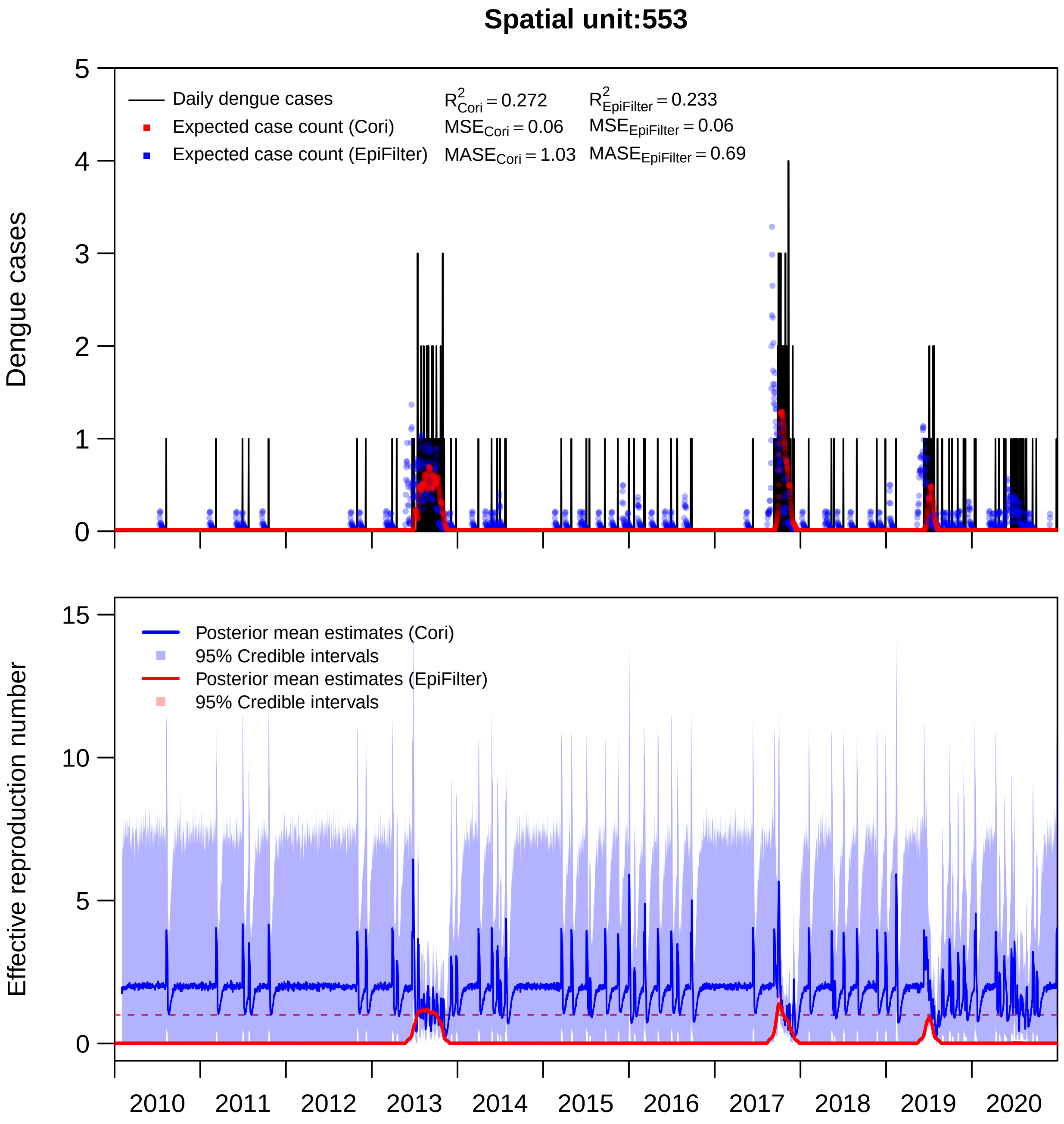

Spatial unit:553
Daily dengue cases
Expected case count (Cori)
Expected case count (EpiFilter)
$R^2_{Cori} = 0.272$
$MSE_{Cori} = 0.06$
$MASE_{Cori} = 1.03$
$R^2_{EpiFilter} = 0.233$
$MSE_{EpiFilter} = 0.06$
$MASE_{EpiFilter} = 0.69$
Dengue cases
0
1
2
3
4
5
Posterior mean estimates (Cori)
95% Credible intervals
Posterior mean estimates (EpiFilter)
95% Credible intervals
Effective reproduction number
0
5
10
15
2010
2011
2012
2013
2014
2015
2016
2017
2018
2019
2020

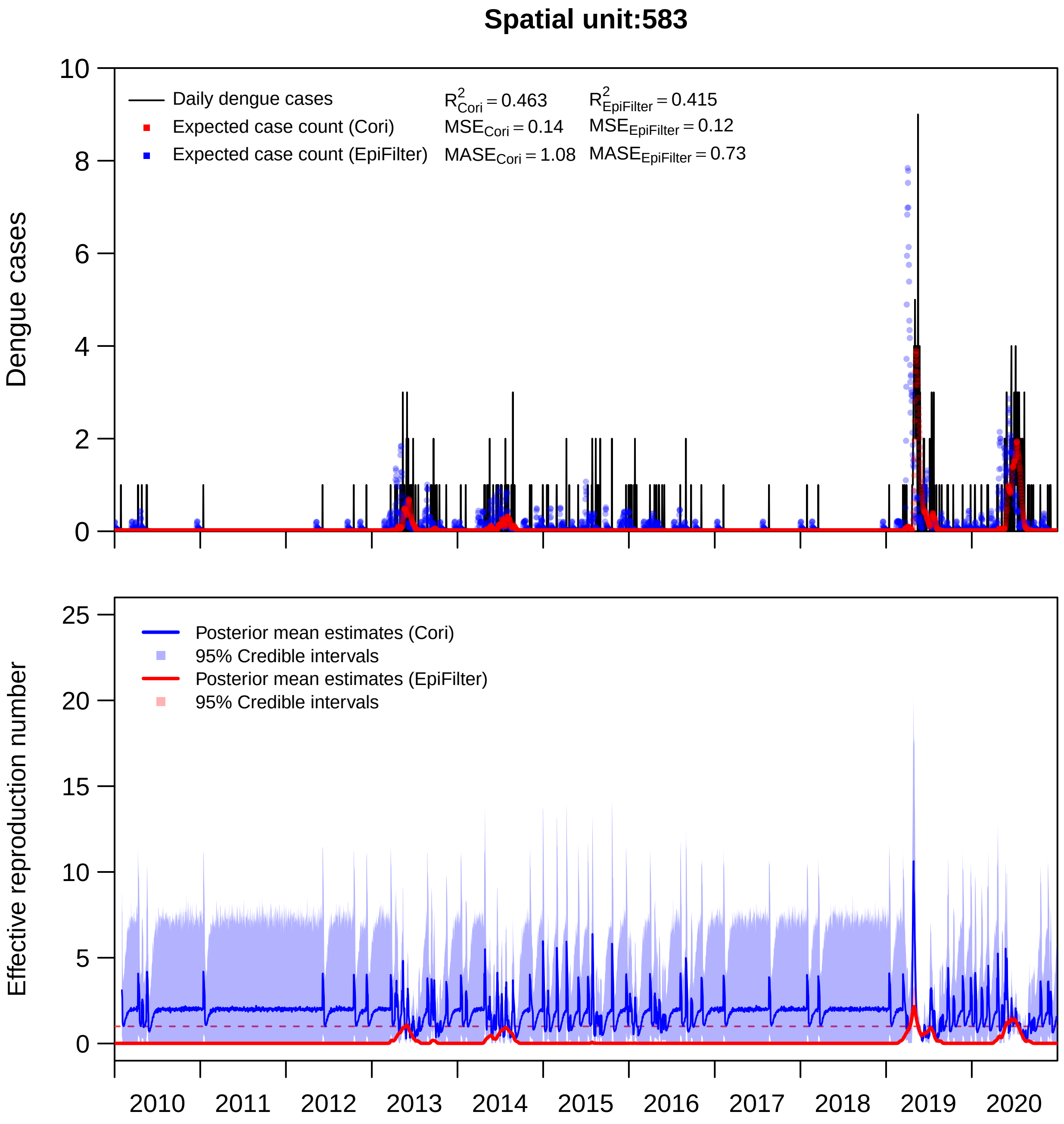

Spatial unit:583
Dengue cases
Daily dengue cases
Expected case count (Cori)
Expected case count (EpiFilter)
$R^2_{Cori} = 0.463$
$MSE_{Cori} = 0.14$
$MASE_{Cori} = 1.08$
$R^2_{EpiFilter} = 0.415$
$MSE_{EpiFilter} = 0.12$
$MASE_{EpiFilter} = 0.73$
0
2
4
6
8
10
Effective reproduction number
Posterior mean estimates (Cori)
95% Credible intervals
Posterior mean estimates (EpiFilter)
95% Credible intervals
0
5
10
15
20
25
2010
2011
2012
2013
2014
2015
2016
2017
2018
2019
2020

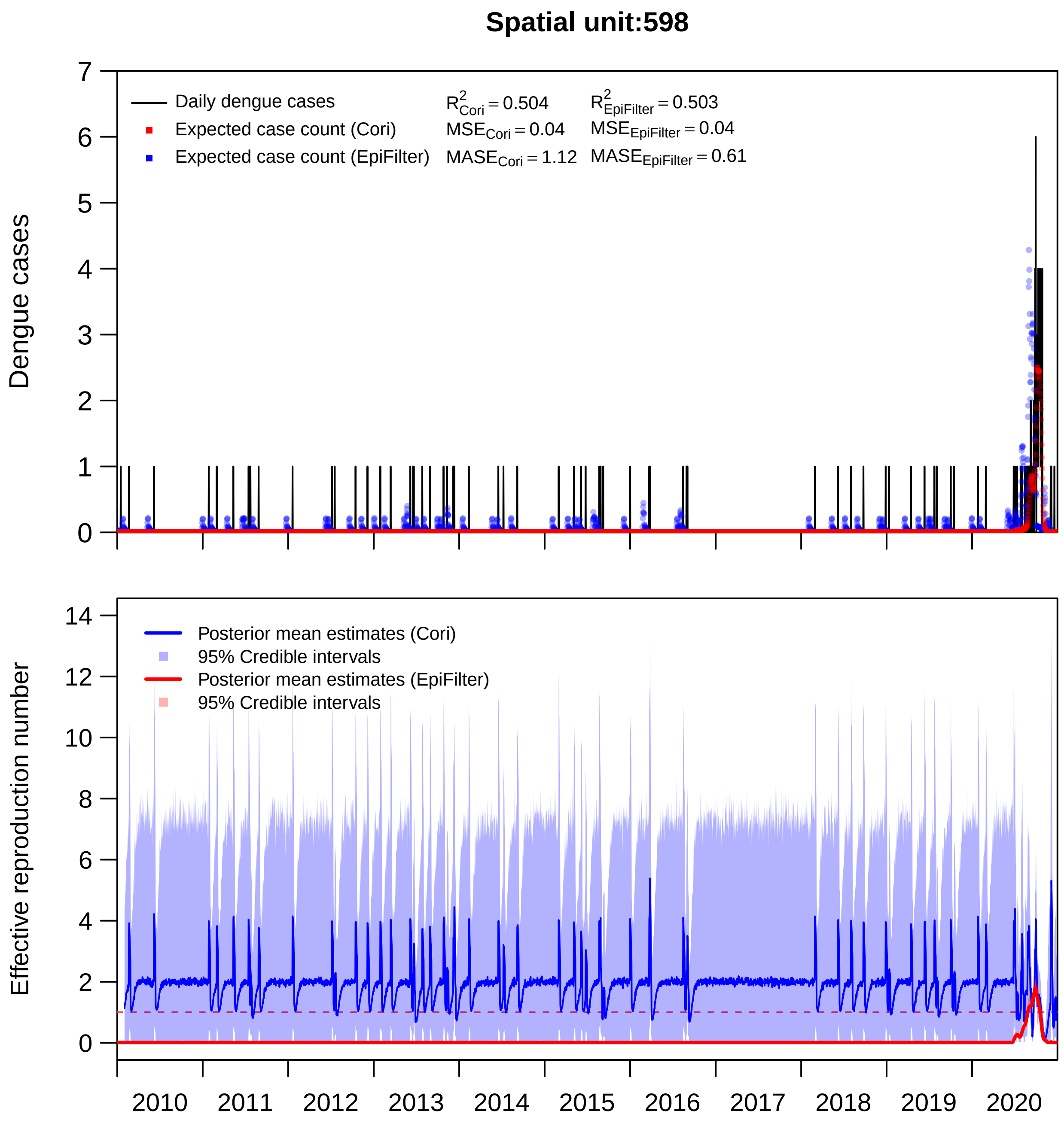

Spatial unit:598
Dengue cases
Daily dengue cases
Expected case count (Cori)
Expected case count (EpiFilter)
$R^2_{Cori} = 0.504$
$MSE_{Cori} = 0.04$
$MASE_{Cori} = 1.12$
$R^2_{EpiFilter} = 0.503$
$MSE_{EpiFilter} = 0.04$
$MASE_{EpiFilter} = 0.61$
Effective reproduction number
Posterior mean estimates (Cori)
95% Credible intervals
Posterior mean estimates (EpiFilter)
95% Credible intervals
2010
2011
2012
2013
2014
2015
2016
2017
2018
2019
2020

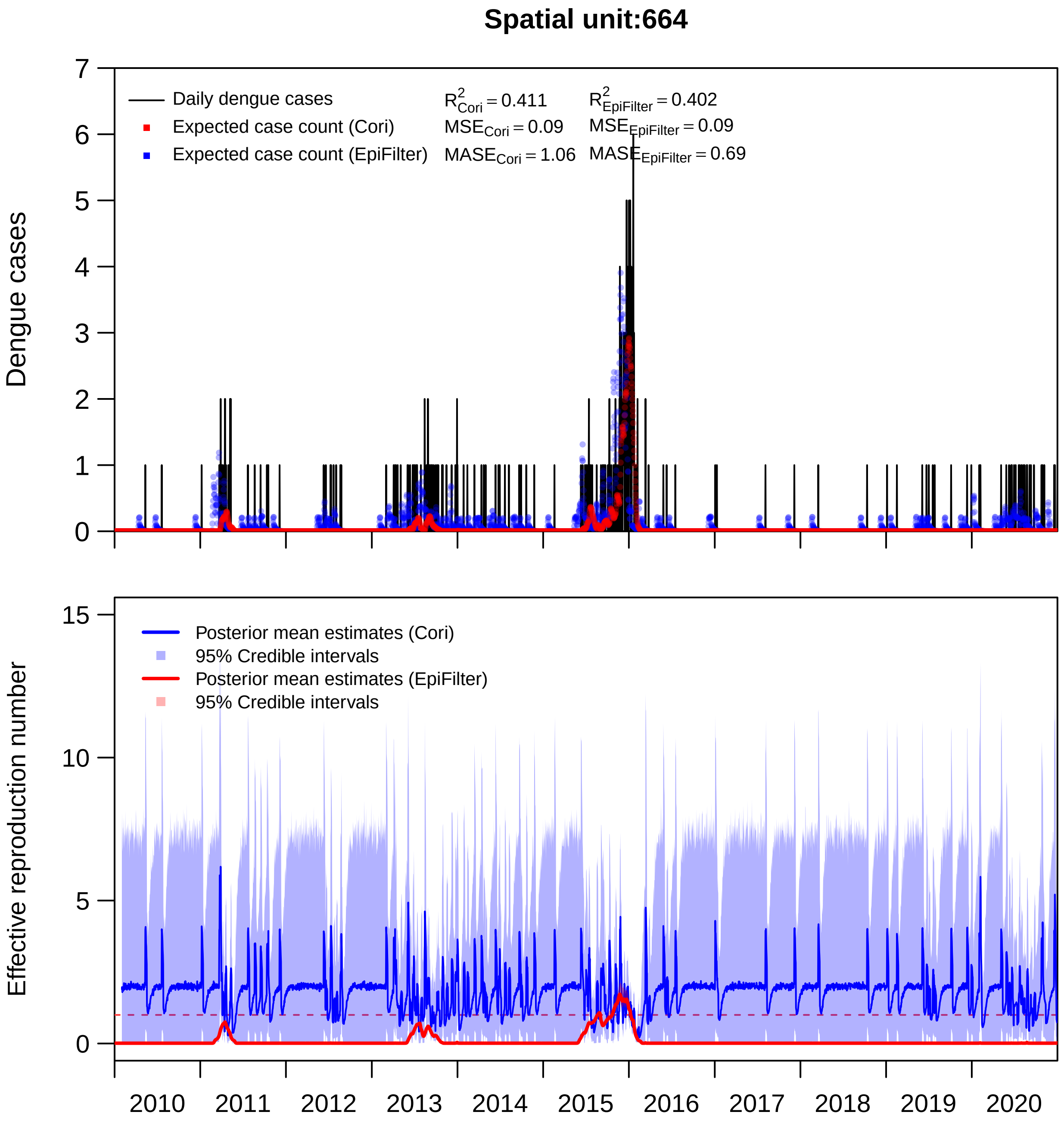

Spatial unit:664
Dengue cases
Daily dengue cases
Expected case count (Cori)
Expected case count (EpiFilter)
$R^2_{Cori} = 0.411$
$MSE_{Cori} = 0.09$
$MASE_{Cori} = 1.06$
$R^2_{EpiFilter} = 0.402$
$MSE_{EpiFilter} = 0.09$
$MASE_{EpiFilter} = 0.69$
0
1
2
3
4
5
6
7
Effective reproduction number
Posterior mean estimates (Cori)
95% Credible intervals
Posterior mean estimates (EpiFilter)
95% Credible intervals
0
5
10
15
2010
2011
2012
2013
2014
2015
2016
2017
2018
2019
2020

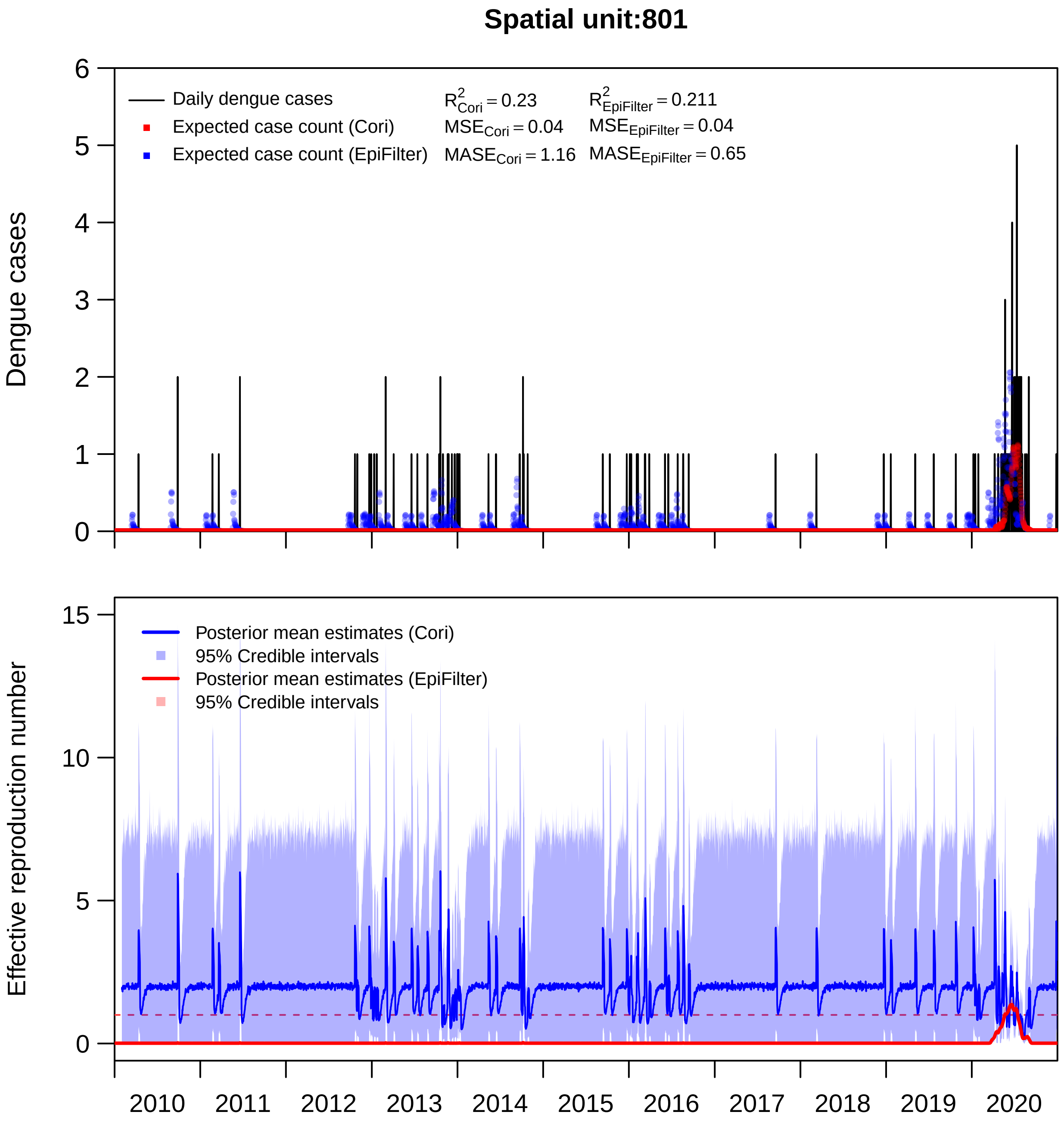

Spatial unit:801
Daily dengue cases
Expected case count (Cori)
Expected case count (EpiFilter)
$R^2_{Cori} = 0.23$
$MSE_{Cori} = 0.04$
$MASE_{Cori} = 1.16$
$R^2_{EpiFilter} = 0.211$
$MSE_{EpiFilter} = 0.04$
$MASE_{EpiFilter} = 0.65$
Dengue cases
Posterior mean estimates (Cori)
95% Credible intervals
Posterior mean estimates (EpiFilter)
95% Credible intervals
Effective reproduction number
2010
2011
2012
2013
2014
2015
2016
2017
2018
2019
2020

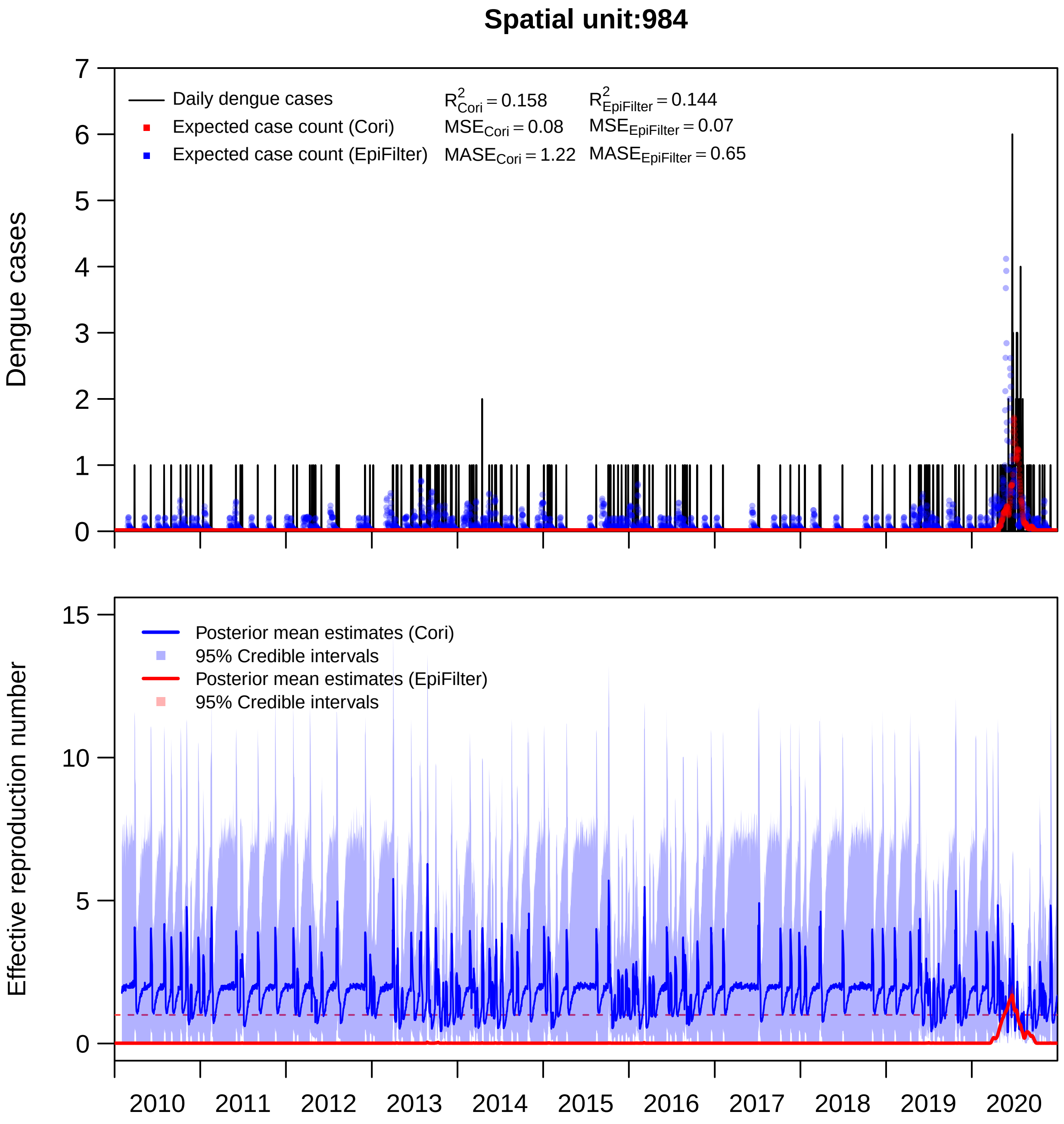

Spatial unit:984
Daily dengue cases
Expected case count (Cori)
Expected case count (EpiFilter)
$R^2_{Cori} = 0.158$
$MSE_{Cori} = 0.08$
$MASE_{Cori} = 1.22$
$R^2_{EpiFilter} = 0.144$
$MSE_{EpiFilter} = 0.07$
$MASE_{EpiFilter} = 0.65$
Dengue cases
0
1
2
3
4
5
6
7
Posterior mean estimates (Cori)
95% Credible intervals
Posterior mean estimates (EpiFilter)
95% Credible intervals
Effective reproduction number
0
5
10
15
2010
2011
2012
2013
2014
2015
2016
2017
2018
2019
2020

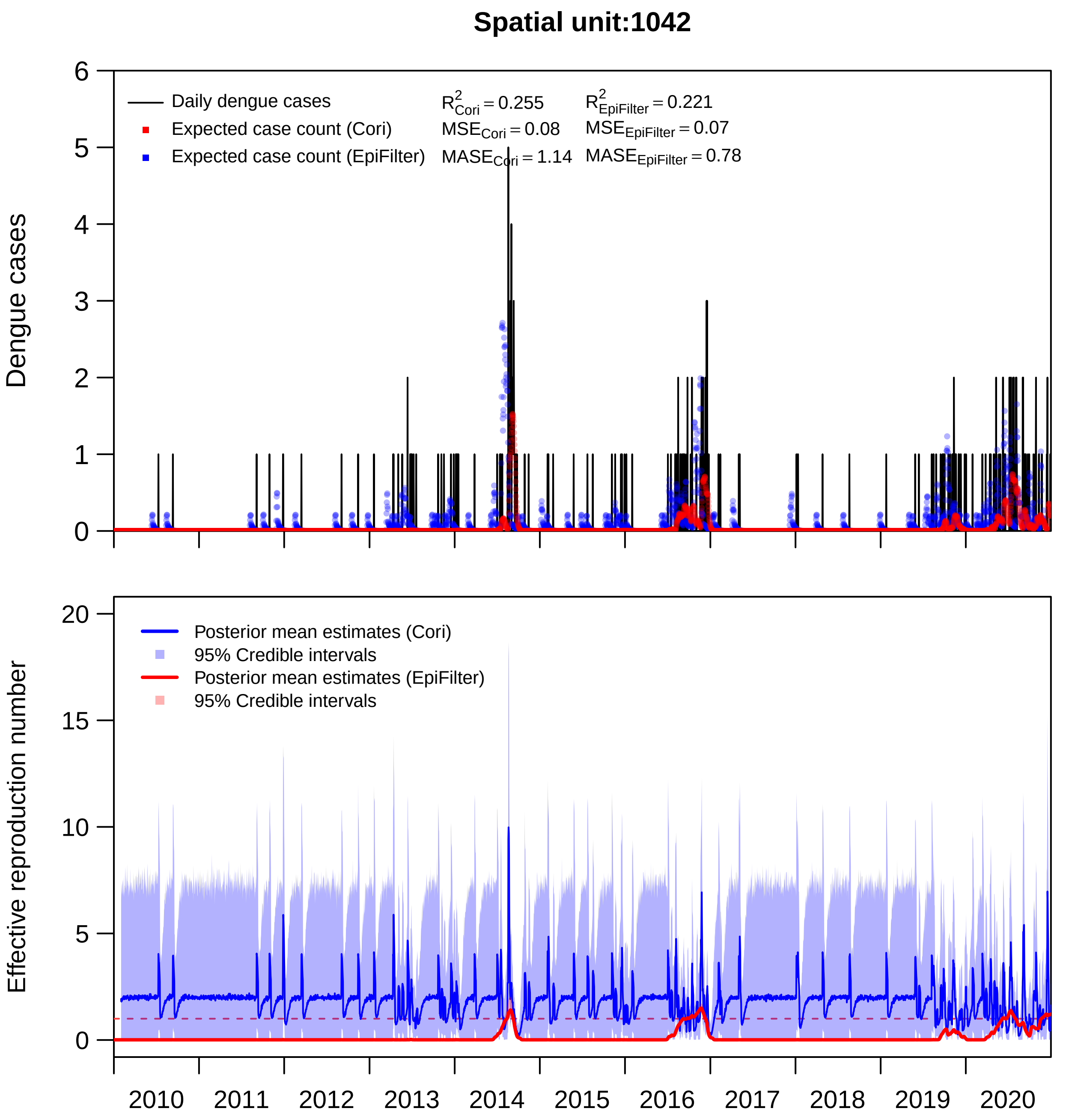

Spatial unit:1042
Daily dengue cases
Expected case count (Cori)
Expected case count (EpiFilter)
$R^2_{Cori} = 0.255$
$MSE_{Cori} = 0.08$
$MASE_{Cori} = 1.14$
$R^2_{EpiFilter} = 0.221$
$MSE_{EpiFilter} = 0.07$
$MASE_{EpiFilter} = 0.78$
Dengue cases
0
1
2
3
4
5
6
Posterior mean estimates (Cori)
95% Credible intervals
Posterior mean estimates (EpiFilter)
95% Credible intervals
Effective reproduction number
0
5
10
15
20
2010
2011
2012
2013
2014
2015
2016
2017
2018
2019
2020

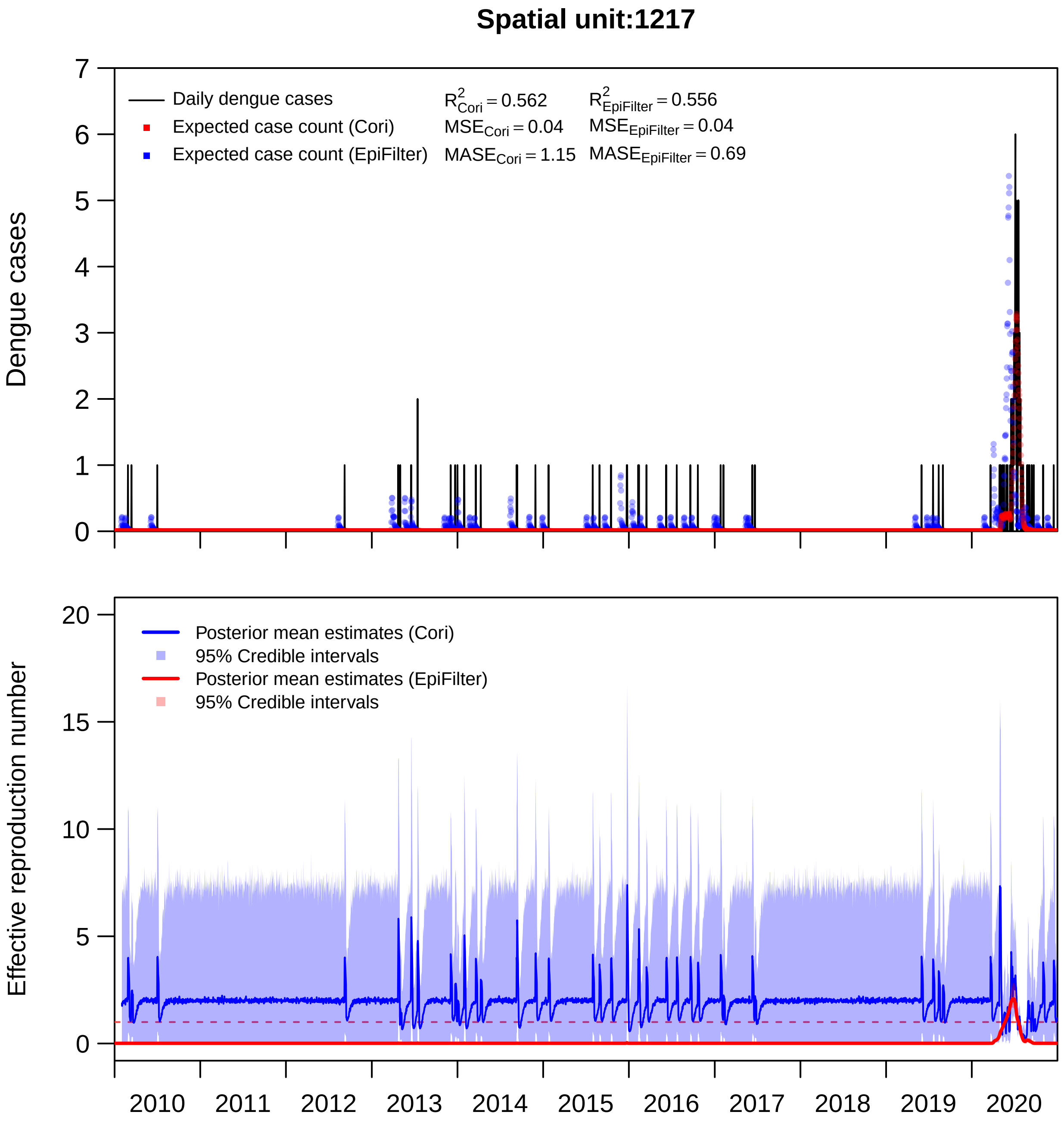

Spatial unit:1217
Dengue cases
Daily dengue cases
Expected case count (Cori)
Expected case count (EpiFilter)
$R^2_{Cori} = 0.562$
$MSE_{Cori} = 0.04$
$MASE_{Cori} = 1.15$
$R^2_{EpiFilter} = 0.556$
$MSE_{EpiFilter} = 0.04$
$MASE_{EpiFilter} = 0.69$
0
1
2
3
4
5
6
7
Effective reproduction number
Posterior mean estimates (Cori)
95% Credible intervals
Posterior mean estimates (EpiFilter)
95% Credible intervals
0
5
10
15
20
2010
2011
2012
2013
2014
2015
2016
2017
2018
2019
2020
